# Broadband extreme ultraviolet zeroth order scatterometry for nanostructure metrology

**DOI:** 10.1038/s41467-026-73052-w

**Published:** 2026-05-19

**Authors:** Francesco Corazza, Emmanouil Kechaoglou, Leo Guery, Zhonghui Nie, Parikshit Phadke, Carl S. Lehmann, Roland Bliem, Peter M. Kraus

**Affiliations:** 1 Advanced Research Center for Nanolithography, Amsterdam, The Netherlands; 2 Van der Waals-Zeeman Institute, Institute of Physics, University of Amsterdam, Amsterdam, The Netherlands; 3 Department of Physics and Astronomy, and LaserLaB, Vrije Universiteit, Amsterdam, The Netherlands

**Keywords:** High-harmonic generation, Characterization and analytical techniques, Optical spectroscopy

## Abstract

The continuous shrinkage of critical dimensions in nanofabrication demands nanometrology at the relevant resolution, which can be achieved using short-wavelength light sources. Most industrial metrology uses periodic structures for process control, exploiting diffraction to probe the fabrication quality of actual device structures. Here, we introduce a table-top high-harmonic generation extreme-ultraviolet scatterometry with broadband illumination. Our method exploits the spectrally resolved 0^th^ diffraction order of the extreme-ultraviolet light, which, while lacking spatial-encoded information, carries valuable spectral information and offers high diffraction efficiency. The use of relative reflectivity removes the need for absolute calibration, and rigorous coupled-wave analysis simulations underpin a library-based reconstruction approach, yielding single-nanometer accuracy for groove height and 10 nm accuracy for critical dimensions. Our work demonstrates broadband extreme-ultraviolet high-harmonic-generation 0^th^ order scatterometry that delivers fast, reliable, non-destructive metrology for structures with at-wavelength features, providing sensitivity and accuracy for details far below the diffraction limit.

## Abstract (web summary)

Scatterometry techniques at extreme ultraviolet wavelength are a promising way to achieve spatially resolved material characterisation. Here, the authors present a scatterometry method based on high-harmonic-generation together with model based reconstruction providing nanometer-level accuracy.

## Introduction

The recent development of nanostructure fabrication has significantly advanced both science and technology^1–3^, and nowadays constitutes the core of high-performance processors. The current state-of-the-art fabrication techniques rely on nanolithography. Interference lithography research tools have achieved reliable patterning of features down to 5 nm on Silicon wafers^4^. In high-volume manufacturing the newest node release is called the 2 nm technology node^5,6^, which has metal pitches of about 20 nm and contacted gate pitches of about 45 nm (the “2 nm” in the name itself does not relate to a relevant feature length). These extremely small dimensions push conventional optical inspection methods to their limits and require dedicated metrology techniques to ensure fabrication quality and consistency at the nanoscale.

The fabrication process control commonly employs characterization techniques such as Scanning Electron Microscopy (SEM)^7–9^, Atomic Force Microscopy (AFM)^10–12^, and optical profilometry^13–15^. However, these methods can pose a challenge for in-line inspection during nanofabrication due to their low acquisition speed and potential to induce wafer damage. Furthermore, most of these approaches are only sensitive to the sample surface, leaving three-dimensional characterization of complex structures an open challenge.

Scatterometry offers an alternative path to conventional imaging-based metrology techniques^16,17^, enabling precise characterization of nanoscale structures. The evaluation of the diffraction patterns created by light scattered off periodic samples enables the extraction of detailed structural information below the diffraction limit. By solving the inverse scattering problem, it becomes possible to reconstruct the morphology of the sample with sub-wavelength detail^18–23^. For this reason, scatterometry is highly useful both in fundamental research and industrial metrology applications as three-dimensional profilometry, where precision and non-invasive analysis are essential. An example is Optical Critical Dimension (OCD) metrology, widely deployed in semiconductor manufacturing in the visible range^24,25^. In practice, OCD inverts angle/polarization/wavelength-dependent diffraction to access sub-diffraction structural parameters, but its performance can be limited by parameter correlations and stack-dependent sensitivities. Furthermore, small-pitch nanostructures illuminated with visible-UV light don’t produce actual diffraction patterns, effectively requiring complex modeling.

The use of short wavelengths in scatterometry, such as extreme ultraviolet (XUV) or soft X-ray radiation, significantly enhances sensitivity to feature sizes. At these wavelengths, single scattering is typically dominant and offers modest correlations between parameters, enabling simultaneous and reliable characterization of multiple structural properties, even for complex geometries^20,26–29^. In the framework of XUV scatterometry performed with broadband illumination, higher diffraction orders are often favored, as they provide spatially encoded information within the diffraction pattern. Here we utilize the 0th order and show that similar structural information can be obtained from its analysis. The 0th order carries spectrally encoded information and can provide the largest diffraction efficiency compared to higher orders. Together with the high brightness of High-Harmonic Generation (HHG), this can be exploited to extract morphological information from a nanostructured sample with high signal-to-noise ratio and fast acquisition, highlighting its potential in nanoscale metrology.

In this work, we present a spectroscopic scatterometry method based on the 0th diffraction order of broadband HHG radiation^30,31^ as a precise and non-destructive metrology tool for nanostructures. By measuring the relative reflectivity, we exploit spectrally encoded information while avoiding the need for absolute calibration. We show that combining planar and conical diffraction geometries provides complementary sensitivity to the grating morphology, enabling the decoupling of critical dimension and groove height contributions in the 0th order signal. The morphology reconstruction is formulated as a library-based inverse problem using Rigorous Coupled-Wave Analysis (RCWA), allowing us to retrieve nanostructure parameters with nanometer-level accuracy. We validate this approach experimentally across a range of feature sizes and demonstrate robust performance down to nanometer-level accuracy.

## Results

The complete morphology (**p**) of a one-dimensional periodic nanostructure is relatively complex and can be described as a collection of parameters:

1 $${{{\bf{p}}}}=\{{{{\rm{pitch}}}},{{{\rm{CD}}}},{{{\rm{GH}}}},{{{\rm{SWA}}}},{{{\rm{LR}}}},{{{\rm{ER}}}},\ldots \}$$

which includes average Critical Dimension (CD), Groove Height (GH), Side-Wall Angle (SWA) and Line Roughness (LR), Edge Roundness (ER) and more. However, for the experiments discussed here, the target designs were chosen to reduce the morphology complexity in good approximation to **p** = {CD, GH}. This simplification is possible by assuming prior knowledge of the pitch, employing a fabrication process that minimizes SWA, and selecting a pitch that is relatively large compared to LR in order to render LR negligible.

The central observable in this work is the relative reflectivity *δR*_*λ*_(**p**), defined as the ratio of the spectrally resolved 0th order diffraction efficiency of the nanostructured target to the reflectivity of an adjacent unstructured Silicon reference on the same substrate:

2 $$\delta {{{{\rm{R}}}}}_{\lambda }({{{\bf{p}}}})=\frac{{{{{\rm{S}}}}}_{\lambda }^{g}({{{\bf{p}}}})}{{{{{\rm{S}}}}}_{\lambda }^{{{{\rm{Si}}}}}}=\frac{{{{{\rm{DE}}}}}_{\lambda }^{{0}^{{{{\rm{th}}}}}}({{{\bf{p}}}})}{{{{{\rm{R}}}}}_{\lambda }^{{{{\rm{Si}}}}}}$$

By normalizing to a local reference acquired in the same measurement, *δR*_*λ*_ is insensitive to absolute instrument response, source spectrum drift, and filter aging, while retaining the morphology-dependent spectral structure that underpins reconstruction.

### Zero order sensitivity to grating morphology

The 0th order relative reflectivity *δR*_*λ*_(**p**) carries morphological information about the grating, but structural parameters such as CD and GH contribute differently to the spectral response depending on the diffraction geometry. To disentangle these two contributions from the 0th diffraction order, each metrology target is designed with two orthogonal line gratings sharing the same morphology, enabling measurements at both *ϕ* = 0^∘^ (planar diffraction) and *ϕ* = 90^∘^ (conical diffraction) without repositioning the sample.

**Fig. 1:**
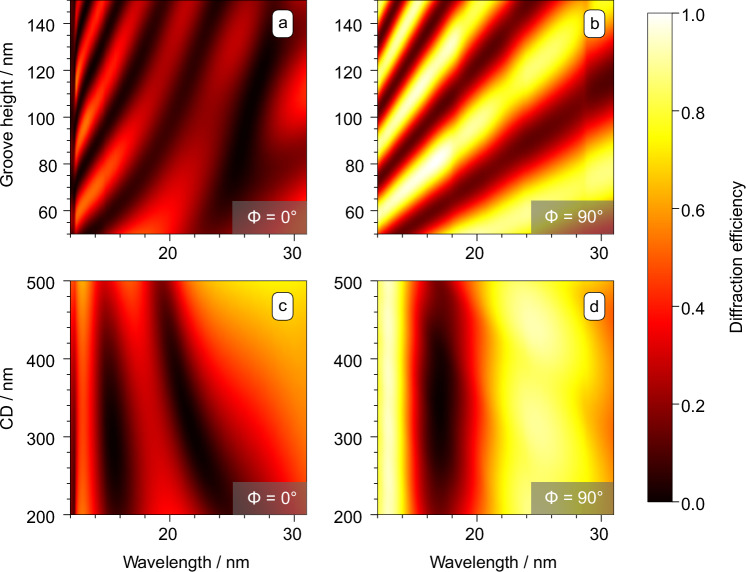
RCWA simulations. Rigorous Coupled-Wave Analysis (RCWA) simulation of the spectrally resolved 0th order diffraction efficiency. **a** 300 nm critical dimension (CD) in planar diffraction conditions (*ϕ* = 0^∘^), **b** 300 nm CD in conical diffraction conditions (*ϕ* = 90^∘^), **c** 80 nm groove height (GH) in planar diffraction conditions (*ϕ* = 0^∘^), **d** 80 nm GH in conical diffraction conditions (*ϕ* = 90^∘^).

We have performed extensive RCWA simulations, which reveal that the two diffraction geometries provide fundamentally different sensitivity to the grating parameters, enabling the independent extraction of CD and GH from the 0th order spectrum. In conical diffraction, the diffraction efficiency is dominated by the phase accumulated over the GH and is largely insensitive to CD, making this geometry ideal for GH reconstruction. In planar diffraction, both parameters contribute to the spectral response, as grating lines at steep incidence partially shadow neighboring lines, introducing a CD-dependent modulation of the interference conditions. This contrast is illustrated in Fig. 1, where the simulated 0th order efficiency is shown as a function of GH and CD for both geometries.

The significance of this approach is illustrated in Fig. 1, which showcases a collection of library items as functions of CDs and GHs. In planar diffraction conditions, Fig. 1a and Fig. 1c, diffraction efficiencies exhibit a dependency on both CD and GH, complicating the independent extraction of these morphological parameters from a single 0th order spectrum. Conversely, under conical diffraction conditions the diffraction efficiency profiles are largely independent of CD, as shown in Fig. 1d, while the plot in Fig. 1b highlights a strong dependence on GH, making this configuration ideal for GH reconstruction.

**Fig. 2:**
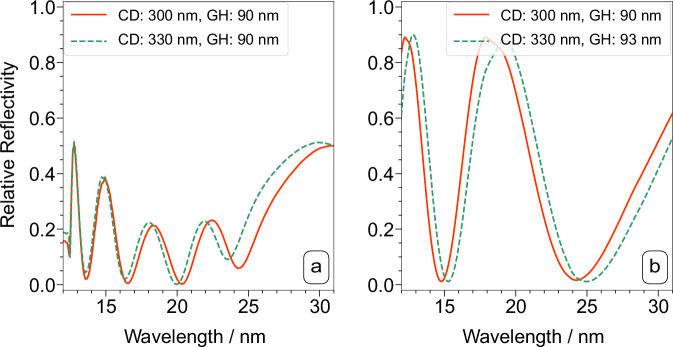
Simulated spectral changes. Comparison of Rigorous Coupled-Wave Analysis (RCWA) simulated relative reflectivity spectra for small variations in morphology. **a** Planar diffraction: comparison of two structures differing by 30 nm in critical dimension (CD). **b** Conical diffraction: comparison of two structures differing by 3 nm in groove height (GH).

This contrast in parameter sensitivity directly impacts the observable spectral response, such that small variations in morphology produce distinct and measurable changes in the reflectivity spectra, providing the basis for morphology reconstruction from the 0th order signal. The practical implications of this sensitivity are illustrated in Fig. 2, which shows that the approach described here is sensitive to the nanostructure morphology in high detail. For example, for a morphology difference of 3 nm in GH (Fig. 2b), the RCWA simulated spectra exhibit a substantial shift and spectral profile deformation, easily detectable with a conventional grating-based XUV spectrometer. A similar conclusion can be drawn from the planar diffraction regime (Fig. 2a) to evaluate the impact of the CD. However, the sensitivity is lower than that for the GH, typically by an order of magnitude. In the context of the experiments presented here, reaching at and below single nanometer CD precision would require additional information from other diffraction channels, for example, spectrally resolved higher diffraction order.

### Broadband scatterometry measurements

The HHG-driven 0th order scatterometry experiment measured 36 different metrology targets with 6 different GHs and 3 different CD, 2 replicas of each target. Each scatterometry target consists of two nanoscale line gratings, that share the same morphology but with two orthogonal periodicity directions (see “Methods”).

**Fig. 3:**
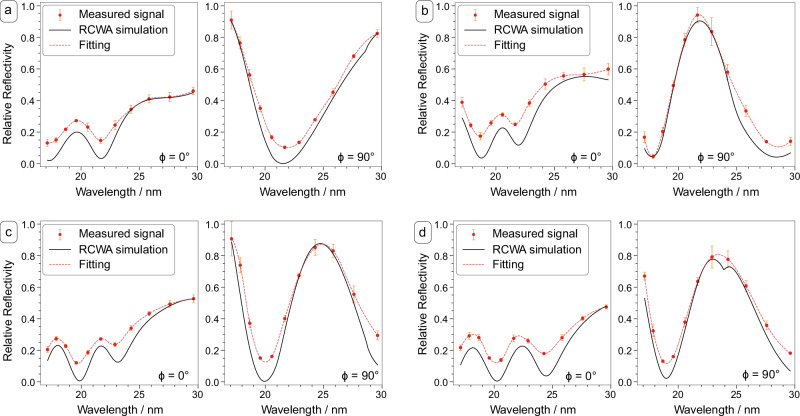
Relative reflectivity measurements for 700 nm pitch targets. Measured relative reflectivity spectra compared with Rigorous Coupled-Wave Analysis (RCWA) simulations for planar and conical diffraction geometries. **a** Sample with 300 nm critical dimension (CD) and 80 nm groove height (GH). **b** Sample with 350 nm CD and 108 nm GH. **c** Sample with 400 nm CD and 92 nm GH. **d** Sample with 400 nm CD and 116 nm GH.

The measurements of relative reflectivity for 4 of the targets are shown in Fig. 3, from which it emerges that the measurements match well with the simulations and are a suitable input to perform morphology reconstruction.

**Fig. 4:**
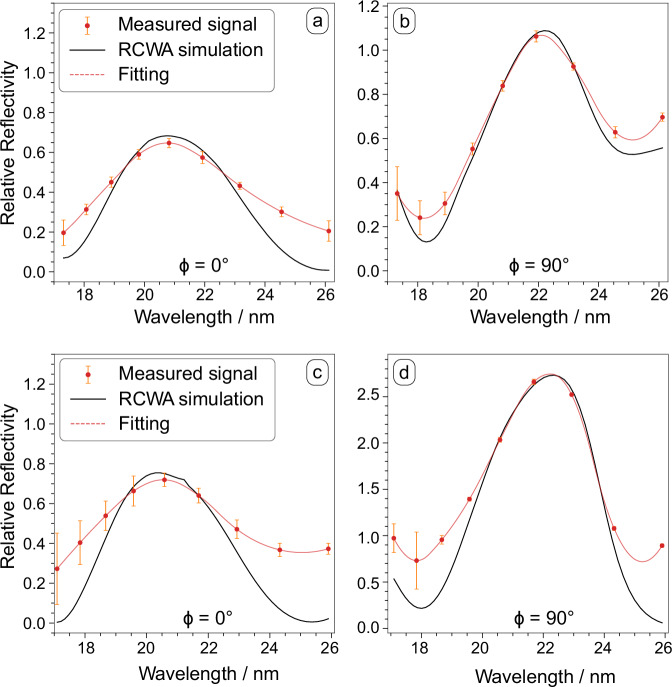
Relative reflectivity measurements for 100 and 200 nm pitch targets. Measured relative reflectivity spectra compared with Rigorous Coupled-Wave Analysis (RCWA) simulations for two metrology targets. **a**, **b** Planar and conical diffraction measurements for a target with 200 nm pitch and 100 nm critical dimension (CD). **c**, **d** Planar and conical diffraction measurements for a target with 100 nm pitch and 50 nm CD. Both targets have a groove height (GH) of 68 nm.

Furthermore, we performed additional measurements on gratings with pitches of 200 nm and 100 nm, and CDs of 100 nm and 50 nm, respectively, in order to evaluate the reconstruction capabilities of this approach at reduced feature sizes. These feature sizes approach the smallest available sizes in standard electron-beam-lithography nanofabrication, which was employed to produce these structures. Figure 4 shows the relative reflectivity spectra measured on representative targets for each pitch, acquired in both planar and conical diffraction geometries and compared to RCWA simulations. These measurements were collected at a reduced incidence angle of 70^∘^, as the sensitivity to transverse features is relatively low at 78^∘^ for these smaller feature sizes. Despite the substantially smaller pitch, the measured spectra exhibit clear and reproducible wavelength-dependent modulations across the probed spectral range.

These results demonstrate that the spectral response provides sufficient sensitivity to resolve nanometer-scale variations in morphology, forming the basis for the experimental reconstruction approach presented below.

### Morphology reconstruction of nanometer-scale features

The reconstruction of the sample morphology **p** from the spectral diffraction efficiency of the 0th order is stated as an inverse problem, and it is addressed with a Least-square Regression (LSR) approach described in detail in the “Methods” section. To assess reconstruction accuracy and validate the confidence intervals returned by our morphology reconstruction routine, we adopt a *χ*^2^-based statistical framework rooted in weighted LSR. For the quantification of the reconstruction accuracy, we compare the reconstructed values *p*_LSR_ to independent reference measurements *p*_ref_ obtained from AFM (for GH) and SEM (for CD). We define the reconstruction bias as:

3 $$b={p}_{{{{\rm{LSR}}}}}-{p}_{{{{\rm{ref}}}}},$$

which provides a direct measure of deviations from the reference metrology. To evaluate the statistical consistency of the *χ*^2^-derived confidence intervals, we account for both the morphology reconstruction uncertainty and the reference uncertainty by introducing the combined standard deviation:

4 $${\sigma }_{c}=\sqrt{{\sigma }_{{{{\rm{LSR}}}}}^{2}+{\sigma }_{{{{\rm{ref}}}}}^{2}},$$

where *σ*_LSR_ is extracted from the confidence interval as *σ*_LSR_ ≈ (*Δp*^−^ + *Δp*^+^)/2, and *σ*_ref_ is the reported uncertainty of the benchmark target characterization. Furthermore, we define the normalized error (or *z*-score) as:

5 $$z=\frac{b}{{\sigma }_{c}},$$

which quantifies the bias in measures of *σ*_*c*_, and indicates whether the reconstruction error is consistent with the predicted uncertainty. This provides an empirical coverage test of the reported confidence intervals. In this context, if the uncertainty model is well calibrated and the residual statistics are approximately Gaussian, the distribution of *z* is expected to be centered around zero, with approximately 68% of points satisfying ∣*z*∣ ≤ 1 and 95% satisfying ∣*z*∣ ≤ 2.

**Fig. 5:**
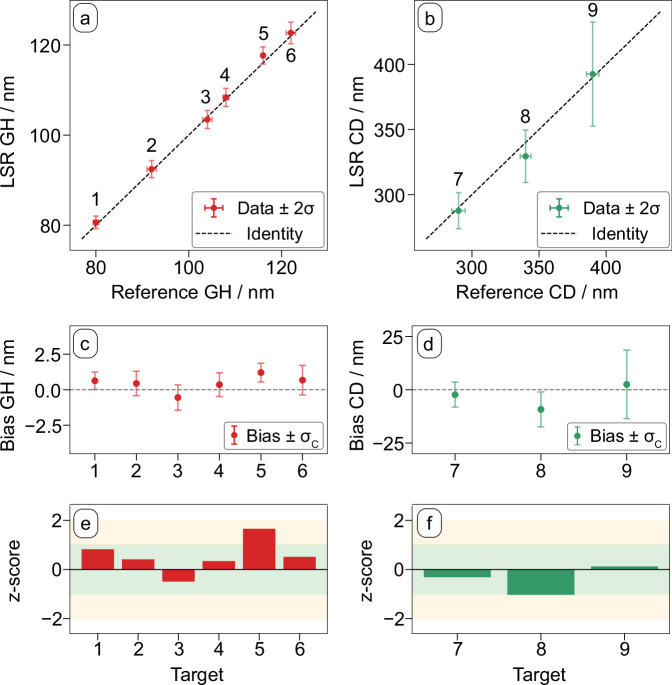
Reconstruction accuracy and uncertainty assessment. **a** Groove height (GH) reconstruction compared with reference atomic force microscopy (AFM) measurements. Error bars represent ±2*σ*. The dashed line indicates perfect agreement. **b** Critical dimension (CD) reconstruction compared with reference scanning electron microscopy (SEM) measurements. **c**, **d** Reconstruction bias for GH and CD, respectively, with error bars given by the combined standard deviation. **e**, **f** Normalized reconstruction error for GH and CD, respectively. Shaded bands indicate the 1*σ* (green) and 2*σ* (orange) confidence intervals. Labels 1–9 identify different metrology targets, all with 700 nm pitch. Full data are provided in the Supplementary Information.

Figure 5 summarizes both reconstruction accuracy and uncertainty consistency by comparing the LSR morphology reconstructions against independent benchmark metrology. Figure 5a, b show the reconstructed values of GH and CD for 9 different metrology targets, plotted against their independent reference values. Here, the error bars correspond to the ±2*σ* uncertainty obtained from the 95% confidence intervals of the least-square inference, while the identity lines represent perfect reconstruction. Figure 5c, d recast these results in terms of reconstruction bias (Eq. (3)), with uncertainties given by *σ*_*c*_ (Eq. (4)). The bias distributions remain centered close to zero and show no clear systematic trend, indicating good agreement with the reference metrology. The normalized errors are reported in Fig. 5e, f, where the shaded bands provide an intuitive visual assessment of the statistical consistency of the reported confidence intervals.

For clarity, Fig. 5 displays only a representative subset of the full dataset, whereas the full validation comprises 36 metrology targets, for which both GH and CD are reconstructed. The complete reconstruction data can be found in the supplementary information in the Supplementary Tables 5 and 6.

Morphology reconstruction was also performed for the four small-pitch targets using the same library-based least-squares regression. For the 200 nm pitch targets, the reconstructed GHs agree with the reference AFM values within 1.1 nm, with combined uncertainties below 1.3 nm. The corresponding CDs are reconstructed within 5–10 nm of the SEM reference values, consistent with the performance observed for the 700 nm pitch dataset.

For the 100 nm pitch targets, GH reconstruction remains accurate at the nanometer level, with deviations from the reference values around 2 nm. The reconstruction of the CD yields 3–7 nm (absolute) bias values, with uncertainty intervals that remain statistically consistent with the observed deviations. Detailed numbers can be found in the supplementary information in the Supplementary Tables 1–4.

### Organic residue contamination

**Fig. 6:**
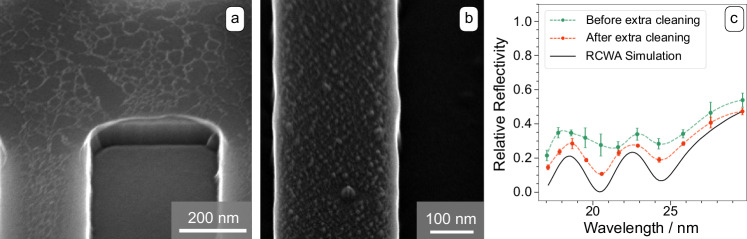
Residue layer. Characterization of the impact of resist residues on the nanostructures. **a** Scanning electron microscopy (SEM) image at 45^∘^ tilt of an etched trench after resist stripping and before additional cleaning. A thin residue layer (light gray) is visible around the etched region. **b** SEM image after additional cleaning, showing residual material as small grains. **c** Comparison of measured relative reflectivity spectra before and after the additional cleaning step.

Despite the overall agreement between experiment and simulation, a systematic mismatch between measured and simulated relative reflectivity can be observed in Fig. 3. We traced this discrepancy to a thin carbonaceous residue left after resist stripping, distributed uniformly over the nanostructured regions. This residue was first identified through tilted SEM imaging (Fig. 6a), while initial XPS measurements were only focused on Si-2p and O-1s for oxide characterization. Once the residue was recognized in SEM, an additional RCA + acid piranha cleaning step was carried out, followed by XPS measurements targeting the C-1s region. All scatterometry results and morphology reconstructions reported in this manuscript were performed after this extra cleaning step. Pre-cleaning measurements are shown only in Fig. 6c as a comparison illustrating the influence of the residue on the spectral response.

The extra cleaning procedure proved effective in reducing the persistent resist layer, as characterized by means of XPS. The results showed that the contamination consists of Carbon, as expected from resist residues, with an average thickness of 0.1 nm, in the form of fractional monolayers. The Carbon residue thickness was measured by comparing the ratio of C-1s and Si-2p atomic percentages. Furthermore, the C-1s region and Auger signals from the XPS analysis showed the presence of both sp^2^ and sp^3^ hybridized species in the C film.

Figure 6c shows the residue layer and its impact on the Relative Reflectivity measurements. From the data shown, it emerges that the residue thickness plays an important role in the local reflectivity of the grating and on the interference conditions that underlie the diffraction process. These results illustrate the exceptional sensitivity of scatterometry to thin surface layers.

## Discussion

The reconstruction capability demonstrated in this work originates from the geometry-dependent sensitivity of the 0th order signal. By combining planar and conical diffraction configurations, the contributions of CD and GH are effectively decoupled, transforming an otherwise correlated inverse problem into a separable one.

It is important to note that the morphology reconstruction is performed by comparing the measured reflectivity spectra against a discrete RCWA library, defined on a finite parameter grid. Therefore, the library sampling determines the numerical resolution of the brute-force search and sets the minimum spacing between candidate geometries. However, this does not constitute a limit as the reconstruction uncertainty is not directly connected to the library mesh size. Instead, it is extracted from the *χ*^2^ loss landscape through confidence intervals derived from the condition *Δχ*^2^ = 1 (for a single parameter), which reflects the curvature of the loss function and the effective noise level of the measurement. This implies that the confidence intervals provide an intrinsic uncertainty estimate of the least-square inference that can be smaller or larger than the grid spacing, which mainly depends on the local sensitivity of the *δ*R_*λ*_(**p**) signal. This approach enables statistically meaningful uncertainty quantification, which is subsequently validated against independent benchmark metrology measurements.

Based on this validation framework, statistically grounded claims can be made regarding both reconstruction accuracy and uncertainty calibration. For GH, the median absolute reconstruction error is 0.6 nm, with the 68th percentile below 0.9 nm and the 95th percentile below 2.6nm, demonstrating nanometer-level accuracy across the full dataset. For CD, the median absolute error is 6.5 nm, with the 68th percentile at 8.5 nm and the 95th percentile at 16.7 nm, while 78.8% of reconstructions fall within a 10 nm error band. Uncertainty validity can be assessed through the normalized-error statistics: for GH, 74.3% of reconstructions satisfy ∣*z*∣ ≤ 1 and 89.6% satisfy ∣*z*∣ ≤ 2, while for CD 74.3% satisfy ∣*z*∣ ≤ 1 and 91.4% satisfy ∣*z*∣ ≤ 2. These results indicate that the *χ*^2^ confidence intervals provide a physically meaningful estimate of reconstruction uncertainty. Overall, this quantification supports typical reconstruction accuracies of 1 nm for GH and 10 nm for CD, validated against independent metrology benchmarks.

Additionally, the 200 nm and 100 nm pitch targets confirm that broadband 0th order XUV scatterometry remains applicable at substantially reduced dimensions, with performance consistent with the morphology reconstruction of the nanostructures with 700 nm pitch.

From a practical point of view, the CD reconstruction accuracy turns out to be an order of magnitude worse than GH. In-plane feature extraction using the 0th diffraction order only is more complicated than for out-of-plane features, because the computation of the Euclidean distances in the *ϕ* = 0^∘^ regime are significantly more sensitive to the amplitude of the signal modulations than in the *ϕ* = 90^∘^, collecting larger uncertainty from parameters not modeled in the simulations, such as post-fabrication residues and surface roughness.

The sensitivity of *δR*_*λ*_ to surface conditions is illustrated by the spectral response to a sub-monolayer carbonaceous residue discussed in the “Results” section. This further explains the larger CD reconstruction uncertainty: the *ϕ* = 0^∘^ geometry is more exposed to amplitude perturbations from unmodeled surface contributions than the *ϕ* = 90^∘^ geometry used for GH extraction.

For semiconductor wafer metrology, optical methods generally stand out as the least invasive. In the case of short-wavelength metrology, however, the ionizing nature of the radiation must also be considered. For the hard materials considered here (e.g., Silicon), exposure-induced damage does not play a significant role. In practical applications, after-exposure and after-development (but prior to etching) metrology is performed on developed photoresists, which may continue to be exposed and thus undergo changes in their optical constants as well as thickness shrinkage^32,33^. This does not preclude process-compatible metrology, as such changes can be calibrated and accounted for in the data analysis. Nevertheless, resist-on-Silicon metrology will present an important future challenge to be addressed by scatterometry.

In summary, we have discussed the 0th diffraction order scatterometry technique based on HHG to characterize the morphology of periodic nanostructures beyond the diffraction limit and beyond the need for precise absolute reflectivity calibration of the experimental apparatus. GHs and CDs are reconstructed from the relative reflectivity spectra, with single-nanometer accuracies for out-of-plane features, and 10 nm precision for transverse features, even in the presence of light contamination on the samples. Looking forward, the next natural step is the extension of the morphology reconstruction algorithm to automatic differentiation in the Simulator-Based Inference framework, with the goal of investigating more complex samples with a large parameters space to explore the sensitivity limits of this technique. More broadly, combining this spatial sensitivity with extreme-ultraviolet transient reflectivity could enable functional metrology of electron and lattice dynamics on femto- to attosecond time scales in semiconductors and strongly correlated materials^34–37^.

## Methods

### Fabrication and characterization of metrology targets

**Fig. 7:**
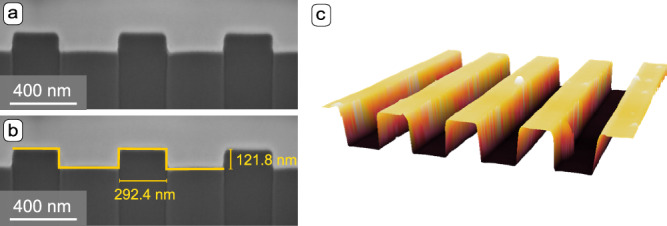
Nanostructured target characterization. **a** Grating cross-section obtained by scanning electron microscopy (SEM). The cross section was prepared using ion-beam-induced deposition followed by ion milling. **b** Geometrical profile fitting using a square line model for parameter extraction. **c** Atomic force microscopy (AFM) measurement (out-of-plane axis not to scale).

**Fig. 8:**
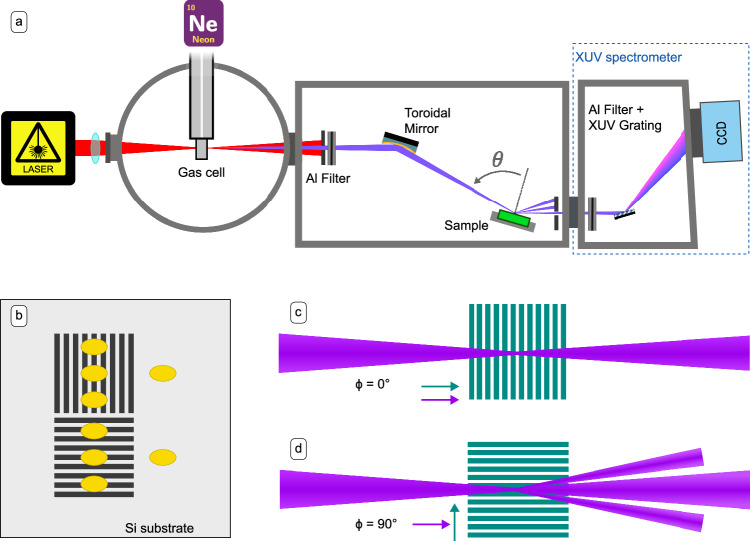
Experimental setup and acquisition procedure. **a** Schematic of the experimental setup. **b** Data acquisition routine, where yellow markers indicate extreme ultraviolet (XUV) beam positions on the sample. **c** Planar diffraction geometry with grating periodicity direction (green arrow) and projected beam propagation direction (violet arrow). **d** Conical diffraction geometry with the same definitions. The azimuthal angle *ϕ* is defined as the angle between these directions.

Each scatterometry target consists of two nanoscale line gratings with a rectangular profile etched into a Silicon substrate, as shown by the cross-section in Fig. 7. These two gratings share the same morphology but with two orthogonal periodicity directions, as shown in (Fig. 8c, d). The samples are designed and fabricated in-house on Silicon via Electron Beam Lithography (see Supplementary Methods). The work presented in the following was carried out on a set of 36 different metrology targets with 700 nm pitch, each of which is written in a 400 × 400 μm^2^ write-field. We have manufactured six samples with six targets each, with three different CDs, two replicas per target. The samples have different GHs between 80 and 130 nm. The morphology **p** of each target was characterized with both AFM and SEM.

After the fabrication process, the samples were characterized via X-Ray Photoelectron Spectroscopy (XPS) which revealed a 1.2 ± 0.1 nm thick native oxide layer on the surface. The thickness was measured by comparing the atomic percent ratios of the native oxide and pure Si signals from the Si-2p core region^38^.

The XPS characterization also detected the presence of some organic contamination in the shape of a fractional monolayer, a leftover from the resist used in the nanofabrication process (more details in the Supplementary Methods).

In addition to the 700 nm pitch targets, a set of 4 nanostructures with pitches of 200 nm and 100 nm was fabricated to probe the applicability of the technique to more aggressively scaled geometries. For each pitch, two independent targets were produced on the same Silicon substrate. We followed the same design and fabrication methodology as for the 700 nm pitch structures, with CDs corresponding to 50% duty cycle.

### HHG-driven scatterometry setup

The experimental setup consists of a laser and a series of vacuum chambers dedicated to the HHG source, focusing of the XUV light onto the sample, and XUV spectrometry, as illustrated in Fig. 8.

**Fig. 9:**
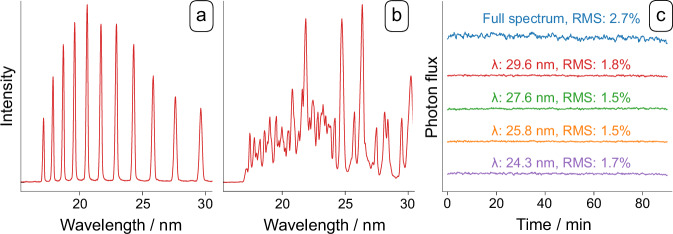
High-harmonic generation (HHG) illumination spectrum. **a** Extreme ultraviolet (XUV) spectrum under discrete harmonic generation conditions. **b** XUV spectrum in a continuum-like regime. **c** Source stability over 90 min in the discrete regime with 2 s exposure time. Stability traces are shown for four individual harmonics and for the total HHG signal.

The primary source is a Ti:Sapphire laser system at 800 nm (Solstice ACE, Spectra Physics), 40 fs pulse duration with a repetition rate of 2 kHz. Approximately 3 W are utilized for High Harmonic Generation. The beam pointing is actively stabilized and focused into a 3 mm long gas cell reaching ~10^15^ W/cm^2^ peak intensity. The cell is supplied with gas by a mass-flow controller that ensures a stable flow and pressure in the generation region. The HHG apparatus can be switched between two configurations to allow some tunability over the XUV spectrum: one dedicated to an ordinary HHG regime, characterized by a discrete harmonics spectrum, and one where the generation conditions are tweaked to obtain a continuum-like spectrum^39^ (see Fig. 9 and Supplementary Methods in Supplementary Fig. 1). For the presented experiments, Neon was chosen as the HHG medium to generate a photon flux in the 11–35 nm wavelength range. The infrared laser light used to drive the HHG process is afterwards filtered out with a pin-hole and a pair of 200 nm thick metallic films made of Aluminum (see Fig. 8).

The samples are mounted in a custom holder onto a 4-axis (*x*, *y*, *z*, *θ*) vacuum compatible stage, with a closed-loop controller.

The HHG beam is focused by a gold-coated toroidal mirror and impinges on the sample with a 78^∘^ ± 0.002^∘^ angle of incidence with respect to the surface normal plane. The reflected 0th diffraction order is sieved out of the diffraction pattern with a pinhole after the sample is subsequently diffracted by a flat-field XUV blazed grating at grazing incidence onto an XUV sensitive CCD with a sensor cooled to a temperature of −90^∘^ C.

A gold-coated toroidal mirror focuses the XUV beam onto the sample. Beam waist characterization was performed in the reflection geometry by using two complementary methods. The first one uses one of the grating targets as the effective edge^40^, and the other one by using a razor blade that was attached at a 7^∘^ angle with respect to the sample surface.

The grating-edge scan provided the beam intensity profile used to extract both beam-waist axes, while a complementary razor-blade scan—limited by its imperfect tangency to the surface—offered a consistency check.

The two methods converged to a short axis (vertical) of 49 ± 3 μm, and to a long axis (horizontal) of 260 ± 7 μm, which is reasonably close to the estimated projection of the beam on the sample at 12^∘^ from grazing incidence.

An estimation of the XUV flux onto the sample is performed by taking into account the recorded spectrum and beam propagation factors. The expression used is:

6 $$\frac{{\phi }_{{{{\rm{XUV}}}}}}{{{{\rm{pulse}}}}}=\frac{{E}_{{{{\rm{meas}}}}}}{{f}_{{{{\rm{propagation}}}}}\,{f}_{{{{\rm{detection}}}}}}=\frac{\frac{{E}_{{{{\rm{total}}}}}}{{\#}_{{{{\rm{pulses}}}}}}}{{T}_{{{{\rm{Al}}}}}\,{R}_{{{{\rm{sample}}}}}\,{R}_{{{{\rm{Col}}}}}\,{\eta }_{{{{\rm{g}}}}}\,{\eta }_{{{{\rm{e-h}}}}}\,{\Phi }_{{{{\rm{CDD}}}}}},$$

where *E*_total_ is the total integrated energy reaching the sensor, *#*_pulses_ is the number of pulses calculated by multiplying the repetition rate of the laser with the total acquisition time for acquisition of a spectrum, *T*_Al_ is the transmission of the second 200 nm Al filter^41^, *R*_*i*_ are the reflectivities of the sample and collection mirror^42^, *η*_g_ is the first order diffraction efficiency of the analyzing grating^43^, *η*_e-h_ is the electron-hole pair creation efficiency of CCD sensors^44^ and *Φ*_CDD_ is the specific quantum efficiency provided by the manufacturer.

The HHG generated in Ne spans well over the Al edge, therefore the estimation should be considered conservative. Nevertheless, a typical spectrum gives us an estimation of approximately $10\frac{{{{\rm{pJ}}}}}{{{{\rm{pulse}}}}}$ which is consistent with previous calculations performed on the setup^45^. Considering the above, the fluence on the samples is calculated under the assumption of an ellipsoidal beam at $100\frac{{{{\rm{nJ}}}}}{{{{{\rm{cm}}}}}^{2}}$.

### Planar and conical diffraction acquisition

Scatterometry measurements are performed by recording the reflected 0^th^ diffraction order from a set of positions on the nanostructured sample and from a pristine flat surface region on the same substrate, as shown in (Fig. 8b). The orientation of the targets was chosen to match the azimuthal angles of *ϕ* = 0^∘^ and *ϕ* = 90^∘^, where *ϕ* is defined as the angle between the target periodicity direction and the beam propagation direction projected on the sample plane. The sample orientation was aligned complying with a 0.3^∘^ tolerance, verified experimentally with a raster scan of the metrology targets. The tolerance was chosen evaluating the impact of misalignment of the azimuthal angle with RCWA simulations. These two configurations correspond to the planar diffraction regime and conical diffraction regime, shown in Fig. 8c and d, respectively. For each target, spectra are averaged over multiple positions on both the structured and unstructured regions to reduce the impact of localized defects and nanoparticles. The averaged spectra are then used to compute the relative reflectivity *δR*_*λ*_(**p**) defined in Eq. (2).

For the sake of comparison and reproducibility, the scatterometry measurements were repeated for both the discrete and the continuum-like HHG regimes, aiming to ensure coherent data overlap for different probing spectral components. The presented experiments were carried out with a 2 s exposure time and by averaging over five spectra per dataset. This choice was made to reduce the impact of spectral fluctuations below the 1% (RMS) threshold. A series of tests showed that shorter exposure times, down to 100 ms, would also provide a sufficient signal-to-noise ratio to enable reconstruction.

### Spectral fitting and relative reflectivity extraction

The raw spectra collected during each measurement are processed to extract the relative reflectivity *δ*R_*λ*_(**p**) defined in Eq. (2), with the specific procedure depending on the HHG regime used. In the case of an optimal HHG process, the XUV spectrum comprises a series of well-defined discrete peaks. Each peak was individually fitted to a Gaussian curve, and the area under the curve within the 1-sigma width was considered as the signal value for each datapoint. From the spectral point of view, each datapoint was located at the center of each Gaussian curve. Consequently, the relative reflectivity of a given target is obtained by averaging the fitted spectra from different locations on the structured target and dividing it by the average substrate reflection, which is a set of averaged spectra from the unstructured Silicon. Variations in reflectivity were taken into account by calculating the statistical variance between different measured positions on each grating.

For quasi-continuous XUV spectra, the relative reflectivity was directly derived by dividing the measured zeroth-order spectrum by that obtained from a pristine Silicon reference surface. A narrow Gaussian low-pass filter was applied in the Fourier space to minimize artifacts caused by rapid signal oscillations while preserving the overall signal integrity. The beyond phase-matching regime usually presents a photon flux and spectral fluctuations which are an order of magnitude worse than the discrete spectrum HHG regime. For this reason, we chose to discuss morphology reconstruction results for the optimal HHG regime, while we only use the nearly continuous spectrum HHG to validate the spline polynomial fitting choice as the routine that best describes the measured relative reflectivity signal (more detail in Supplementary Methods, also in Supplementary Fig. 2).

Ultimately, the relative reflectivity and its measured uncertainty are processed by the reconstruction routine using Eq. (7) to compute the residuals, which are then integrated along the wavelength axis. This produces the parametric function at the center of the optimization problem.

### RCWA simulator

Diffraction from the one-dimensional gratings is modeled under the assumption of a perfectly homogeneous Silicon substrate, with spatially uniform Silicon density (*ρ*_*Si*_), native oxide density (${\rho }_{Si{O}_{2}}$), and complex refractive indices.

The complex refractive indices *n* + *ik* for each material were acquired from the CXRO (Center for X-Ray Optics) database^42,46^.

To model diffraction, we focus on infinitely extended structures with a mono-dimensional periodicity, which feature lines with a rectangular cross-section. However, this is not a fundamental limitation. As established in the “Results” section, the morphology of the grating is simplified to the parameter set **p** = {CD, groove}, always assuming the pitch to be known. This assumption holds in most practical cases, given that lithographic processes are optimized to ensure minimal periodicity errors. Within the framework of linear optics, the simulation of diffraction relies on solving Maxwell’s equations. Our study employs the Rigorous Coupled-Wave Analysis method, a deterministic and non-iterative approach, where the electromagnetic fields are expanded in the grating region into a series of spatial harmonics, enabling the computation of field distributions in both the grating and surrounding media^47,48^. This method is inherently stable, avoiding the numerical instabilities often encountered in alternative approaches, and achieves convergence based solely on the number of modes (2*N* + 1) used in the expansion^49,50^. Furthermore, the method rigorously conserves energy and ensures that the results converge to physically meaningful values as the number of harmonics is increased. After an evaluation of the convergence conditions and the parameter space of interest, we have opted for *N* = 100.

An extensive RCWA-based simulation was conducted to develop a library of diffraction efficiencies over a given spectral range and grating parameters. This library serves as a foundation for reconstructing the nanoscale morphology of the grating structures based on their diffraction signatures. By systematically varying the grating parameters, the simulation captures a comprehensive dataset that correlates specific morphological features with their corresponding diffraction efficiencies. This approach enables the development of robust algorithms to invert the measured diffraction data for precise morphological characterization of nano-structured materials.

### Library-based least-squares morphology reconstruction

Reconstructing the sample morphology **p** from the spectral diffraction efficiency of the 0th order is an inverse problem with no closed-form analytical solution, as no easily invertible model directly maps the measured signal to structural parameters. Equation (2) details how the measured data is processed to express relative reflectivity changes, which are related to the sample morphology. Among the possible methodologies, we implement a library-based approach that contains simulated reflectivity spectra, generated with our RCWA simulator. In general terms, the morphology reconstruction process consists of comparing the measured *δ*R_*λ*_(**p**) to the simulated spectra stored in the library, with the objective of identifying the parameter set that best matches the experimental results. We report a morphology reconstruction approach based on LSR, inspired by published works^21,51^. We also developed an approach based on features tracking to simplify the inverse problem by narrowing down the simulation space in the library search, delivering a computational advantage and faster convergence.

It is possible to exploit the nature of interference to extract sample morphology information without the need to work with a full spectral shape. It can be sufficient to track down some high-interest points, for example, where destructive interference is dominating. Similar ideas were previously implemented in thin-film growth monitoring^52^ and optical ellipsometry^53,54^. The features tracking approach offers a complementary method for reconstructing morphology by analyzing the spectral position of distinct features in the relative reflectivity spectrum, such as local maxima and minima. Although feature tracking does not offer the same precision and robustness as other approaches due to its limited number of spectral points, it remains valuable in specific contexts. One of the main challenges in working with experimental data lies in accurately identifying the true wavelength positions of maxima and minima, as measurement noise and uncertainties introduced by the fitting procedures can lead to deviations. Consequently, this method is less reliable for general nanostructure characterization, but it serves as a supplementary tool by providing a priori information to refine the library search input space. Features tracking was employed in this work as an initial step in the morphology reconstruction process to narrow down search spaces to subsets that closely match the experimental data. Furthermore, features tracking is particularly beneficial when dealing with gratings affected by severe surface contamination or structural defects that significantly alter the sample reflectivity and light interference conditions. In such cases, the LSR approaches are not reliable because of the presence of consistent offsets in the experimental spectra, while the position of minima and maxima generally depends only on the average structure morphology.

In practice, the LSR approach is widely used due to its computational efficiency and well-established mathematical framework^55^. From a data analysis standpoint, one common strategy for implementing LSR involves computing the residuals for each wavelength between the measured relative reflectivity and the items of a simulated library with a set of morphology parameters. The residuals can be defined as:

7 $${\chi }_{\lambda,{{{\bf{p}}}}}^{2}=\frac{{\left[\delta {{{{\rm{R}}}}}_{\lambda }-{{{{\mathscr{F}}}}}_{\lambda,{{{\bf{p}}}}}\right]}^{2}}{{\sigma }_{\lambda,{{{\bf{p}}}}}^{2}}$$

8 $${\sigma }_{\lambda,{{{\bf{p}}}}}^{2}={\sigma }_{\lambda \,(\exp )}^{2}+{\sigma }_{\lambda,{{{\bf{p}}}}\,(sys)}^{2}$$

where ${{{{\mathcal{F}}}}}_{\lambda,{{{\bf{p}}}}}$ represents the simulated relative reflectivity for the **p** morphology and ${\sigma }_{\lambda,{{{\bf{p}}}}}^{2}$ is the variance. The latter is composed of two terms: the first one accounts for the measured uncertainty of the experiments, and the second includes systematic errors, modeled as linearly dependent on the simulated signal^51^. In this case we used ${\sigma }_{\lambda,{{{\bf{p}}}}\,(sys)}^{2}=a\cdot {{{{\mathcal{F}}}}}_{\lambda,{{{\bf{p}}}}}\,+\,b$.

The residuals are weighted by the inverse of the variance. The inverse problem in scatterometry is often framed as a Least Squares Regression problem:

9 $${{{{\bf{p}}}}}_{{{{\rm{LSR}}}}}=\arg {\min }\,{{{{\bf{p}}}}}{\sum }_{\lambda }{\chi }_{\lambda }^{2}$$

Typically, this kind of additional parameters cannot be implemented using LSR, but requires a Maximum Likelihood Estimation (MLE) regression protocol^56^. In the work presented here, we limit the optimization problem to the LSR protocol to prove that 0th order scatterometry can yield precise morphology reconstruction without the need of modeling uncertainty parameters beyond those experimentally measured.

The method minimizes the weighted sum of squared residuals between the measured and simulated relative reflectivity. Its performance therefore depends on the choice of weight factors, which are taken as the inverse of the total variance assigned to each spectral point. Incorrect assumptions about these weights can introduce systematic biases in the reconstructed parameters and have motivated more general inference schemes that explicitly treat nuisance parameters within a MLE framework^51,56,57^.

In the present work, we retain a *χ*^2^-based least-squares formulation, but extend the variance model to include systematic contributions in addition to the experimentally measured noise. This enables us to capture unmodeled uncertainty (linear) contributions while preserving the computational simplicity of the weighted-LSR approach.

To develop a method for reconstructing the nanostructure morphology starting from the diffraction efficiencies, we generated a library containing the simulated relative reflectivity changes. The library was designed to cover a parameter space that includes gratings with GHs ranging from 60 to 150 nm, in 1 nm increments, and CDs between 200 and 500 nm, in 10 nm increments. The entire framework described in this paper, both in simulation and experiments, revolves around TE polarization, with the electric field oriented parallel to the sample surface. Among the key parameters, the incidence angle *θ* was chosen to match experimental conditions, set at *θ* = 78^∘^. The simulations covered the two azimuthal angles *ϕ* = 0^∘^ and *ϕ* = 90^∘^ to match the planar and conical diffraction regimes. These two distinct diffraction conditions are the enabling factor to achieve the separation and independent extraction of CD and GH from the 0th order spectrum.

Diffraction inherently includes the phase accumulated over the line height and the interference of fields within the grating profile while variations in CD act only as a relatively small perturbation on this dominant response. This effect can be exploited to disentangle the CD and GH contributions on the 0th order spectrum. The observed spectral behavior differences are attributed to the rectangular profile of the grating lines and their orientation relative to the plane of incidence. In planar diffraction with steep incident angles, grating lines partially shadow neighboring lines, altering the interference conditions underlying diffraction. This shadowing effect is absent when the grating lines are parallel to the incidence plane, as in *ϕ* = 90^∘^, facilitating more reliable GH measurements.

## Supplementary information

Supplementary Information

Transparent Peer Review file

## Data Availability

The data generated in this study are available under restricted access due to the involvement of long-term collaborations with industrial partners and the presence of potentially sensitive research components. For legal reasons, access to the raw data of the relative reflectivity measurements and RCWA simulations can be obtained by contacting the corresponding author, P.M.K. The datasets will be provided within 4 weeks from the request, without a time limit and subject to compliance with existing collaboration agreements and confidentiality requirements.

## References

[1] Moore, G. E. Cramming more components onto integrated circuits. *Proc. IEEE* **86**, 82–85 (1998).

[2] Priolo, F., Gregorkiewicz, T., Galli, M. & Krauss, T. F. Silicon nanostructures for photonics and photovoltaics. *Nat. Nanotechnol.* **9**, 19–32 (2014). 24390564 10.1038/nnano.2013.271

[3] Waldrop, M. M. The chips are down for Moore’s law. *Nat. N.* **530**, 144 (2016). 10.1038/530144a 26863965

[4] Giannopoulos, I., Mochi, I., Vockenhuber, M., Ekinci, Y. & Kazazis, D. Extreme ultraviolet lithography reaches 5 nm resolution. *Nanoscale* **16**, 15533–15543 (2024). 39133026 10.1039/d4nr01332h

[5] Radhamma, E., Balaji, B., Murthy, A. K. & Naik, R. P. Design analysis and fabrication of finfet using 3 nm technology. In *AIP Conference Proceedings* Vol. 2519 (AIP Publishing, 2022).

[6] Fu, N., Liu, Y., Ma, X. & Chen, Z. EUV lithography: state-of-the-art review. *J. Microelectron. Manuf.* **2**, 1–6 (2019).

[7] Asano, M., Ikeda, T., Koike, T. & Abe, H. Evaluation of producer’s and consumer’s risks in scatterometry and scanning electron microscopy metrology for inline critical dimension metrology. *J. Micro/Nanolithogr. MEMS MOEMS* **5**, 043006–043006 (2006).

[8] Nakamae, K. Electron microscopy in semiconductor inspection. *Meas. Sci. Technol.* **32**, 052003 (2021).

[9] López de la Rosa, F., Sánchez-Reolid, R., Gómez-Sirvent, J. L., Morales, R. & Fernández-Caballero, A. A review on machine and deep learning for semiconductor defect classification in scanning electron microscope images. *Appl. Sci.* **11**, 9508 (2021).

[10] Pou, P. et al. Structure and stability of semiconductor tip apexes for atomic force microscopy. *Nanotechnology* **20**, 264015 (2009). 19509446 10.1088/0957-4484/20/26/264015

[11] Chen, J. & Xu, K. Applications of atomic force microscopy in materials, semiconductors, polymers, and medicine: a minireview. *Instrum. Sci. Technol.* **48**, 667–681 (2020).

[12] Zawierta, M. et al. Atomic force microscopy with integrated on-chip interferometric readout. *Ultramicroscopy* **205**, 75–83 (2019). 31247456 10.1016/j.ultramic.2019.05.011

[13] Chen, L.-C., Duong, D.-H. & Chen, C.-S. Optical 3-D profilometry for measuring semiconductor wafer surfaces with extremely variant reflectivities. *Appl. Sci.* **9**, 2060 (2019).

[14] Deng, F. et al. Three-dimensional surface inspection for semiconductor components with fringe projection profilometry. In *Optical Metrology and Inspection for Industrial Applications IV* Vol. 10023, 175–186 (SPIE, 2016).

[15] Gallagher, J. C. et al. Detecting defects that reduce breakdown voltage using machine learning and optical profilometry. *Sci. Rep.* **14**, 7440 (2024). 38548848 10.1038/s41598-024-57875-5 PMC10978952

[16] Nakasuji, M. et al. Development of coherent extreme-ultraviolet scatterometry microscope with high-order harmonic generation source for extreme-ultraviolet mask inspection and metrology. *Jpn. J. Appl. Phys.* **51**, 06FB09 (2012).

[17] Madsen, M. H. & Hansen, P.-E. Scatterometry—fast and robust measurements of nano-textured surfaces. *Surf. Topography Metrol. Prop.* **4**, 023003 (2016).

[18] Esashi, Y. et al. Tabletop extreme ultraviolet reflectometer for quantitative nanoscale reflectometry, scatterometry, and imaging. *Rev. Sci. Instrum.* **94**, 123705 (2023). 10.1063/5.0175860 38109468

[19] Sherwin, S., Hettermann, M., Houser, D., Long, L. & Naulleau, P. Sub-angstrom critical dimension metrology with euv scatterometry. In *Photomask Technology 2024* Vol. 13216, 132160V (SPIE, 2024).

[20] Porter, C. et al. Soft X-ray: novel metrology for 3D profilometry and device pitch overlay. In *Metrology, Inspection, and Process Control XXXVII* Vol. 12496, 412–420 (SPIE, 2023).

[21] Lohr, L. M. et al. Nanoscale grating characterization using EUV scatterometry and soft X-ray scattering with plasma and synchrotron radiation. *Appl. Opt.* **62**, 117–132 (2022). 10.1364/AO.475566 36606857

[22] Bahrenberg, L. et al. Characterization of nanoscale gratings by spectroscopic reflectometry in the extreme ultraviolet with a stand-alone setup. *Opt. Express* **28**, 20489–20502 (2020). 32680107 10.1364/OE.396001

[23] Gross, H., Rathsfeld, A., Scholze, F. & Bär, M. Profile reconstruction in extreme ultraviolet (EUV) scatterometry: modeling and uncertainty estimates. *Meas. Sci. Technol.* **20**, 105102 (2009).

[24] den Boef, A. J. Optical wafer metrology sensors for process-robust CD and overlay control in semiconductor device manufacturing. *Surf. Topography: Metrol. Prop.* **4**, 023001 (2016).

[25] Germer, T. A., Patrick, H. J., Silver, R. M. & Bunday, B. Developing an uncertainty analysis for optical scatterometry. In *Metrology, Inspection, and Process Control for Microlithography XXIII* Vol. 7272, 255–265 (SPIE, 2009).

[26] Klein, C. et al. Optimized euv scatterometry measurements with tunable high harmonic generation and the Fisher information matrix. In *Metrology, Inspection, and Process Control XXXIX* Vol. 13426, 24–25 (SPIE, 2025).

[27] Barnes, B. M. et al. Lab-based multi-wavelength EUV diffractometry for critical dimension metrology. In *Metrology, Inspection, and Process Control XXXIX* Vol. 13426, 399–413 (SPIE, 2025).

[28] Shao, Y. et al. Wavelength-multiplexed multi-mode euv reflection ptychography based on automatic differentiation. *Light Sci. Appl.* **13**, 196 (2024). 39160154 10.1038/s41377-024-01558-3 PMC11333750

[29] Ku, Y.-S. et al. EUV scatterometer with a high-harmonic-generation EUV source. *Opt. Express* **24**, 28014–28025 (2016). 27906368 10.1364/OE.24.028014

[30] Li, X., l’Huillier, A., Ferray, M., Lompré, L. & Mainfray, G. Multiple-harmonic generation in rare gases at high laser intensity. *Phys. Rev. A* **39**, 5751 (1989). 10.1103/physreva.39.5751 9901157

[31] Krausz, F. & Ivanov, M. Attosecond physics. *Rev. Mod. Phys.* **81**, 163–234 (2009).

[32] Sadegh, N. et al. Xuv induced bleaching of a tin oxo cage photoresist studied by high harmonic absorption spectroscopy. *J. Photopolym. Sci. Technol.* **33**, 145–151 (2020).

[33] Sadegh, N., Evrard, Q., Kraus, P. M. & Brouwer, A. M. Xuv absorption spectroscopy and photoconversion of a tin-oxo cage photoresist. * J. Phys. Chem. C.* **128**, 3965–3974 (2024). 10.1021/acs.jpcc.3c07480 PMC10926160 38476827

[34] Kaplan, C. J. et al. Femtosecond tracking of carrier relaxation in germanium with extreme ultraviolet transient reflectivity. *Phys. Rev. B* **97**, 205202 (2018).

[35] Géneaux, R. et al. Attosecond time-domain measurement of core-level-exciton decay in magnesium oxide. *Phys. Rev. Lett.* **124**, 207401 (2020). 32501089 10.1103/PhysRevLett.124.207401

[36] Jager, M. F. et al. Tracking the insulator-to-metal phase transition in VO2 with few-femtosecond extreme UV transient absorption spectroscopy. *Proc. Natl. Acad. Sci.* **114**, 9558–9563 (2017). 28827356 10.1073/pnas.1707602114 PMC5594684

[37] Zhang, Y. et al. Photoinduced active terahertz metamaterials with nanostructured vanadium dioxide film deposited by sol-gel method. *Opt. Express* **22**, 11070–11078 (2014). 24921805 10.1364/OE.22.011070

[38] Cumpson, P. J. The thickogram: a method for easy film thickness measurement in XPS. *Surf. Interface Anal.* **29**, 403–406 (2000).

[39] Sun, H.-W. et al. Extended phase matching of high harmonic generation by plasma-induced defocusing. *Optica* **4**, 976–981 (2017).

[40] Binnard, M. B. Dense line extreme ultraviolet lithography system with distortion matching https://patents.google.com/patent/US20200326633A1/en US Patent 10,712,671 (2020).

[41] Campbell, T. et al. Dynamics of oxidation of aluminum nanoclusters using variable charge molecular-dynamics simulations on parallel computers. *Phys. Rev. Lett.* **82**, 4866–4869 (1999).

[42] Center for X-Ray Optics (CXRO), L. B. N. L. X-ray interactions with matter (optical constants database). https://henke.lbl.gov/optical_constants/.

[43] Frassetto, F. et al. Compact spectrometer for the analysis of high harmonics content of extreme-ultraviolet free-electron-laser radiation. In *Advances in X-Ray/EUV Optics and Components V* Vol. 7802 (eds Goto, S., Khounsary, A. M. & Morawe, C.) 780209. International Society for Optics and Photonics (SPIE, 2010).

[44] Scholze, F., Rabus, H. & Ulm, G. Mean energy required to produce an electron-hole pair in silicon for photons of energies between 50 and 1500 eV. *J. Appl. Phys.* **84**, 2926–2939 (1998).

[45] van der Geest, M. L. S. et al. Extreme ultraviolet-excited time-resolved luminescence spectroscopy using an ultrafast table-top high-harmonic generation source. *Rev. Sci. Instrum.* **92**, 113004 (2021). 34852522 10.1063/5.0064780

[46] Henke, B. L., Gullikson, E. M. & Davis, J. C. X-ray interactions: photoabsorption, scattering, transmission, and reflection at e= 50-30,000 ev, z= 1-92. *At. Data Nucl. Data Tables* **54**, 181–342 (1993).

[47] Moharam, M., Grann, E. B., Pommet, D. A. & Gaylord, T. Formulation for stable and efficient implementation of the rigorous coupled-wave analysis of binary gratings. *J. Opt. Soc. Am. A* **12**, 1068–1076 (1995).

[48] Lalanne, P. Improved formulation of the coupled-wave method for two-dimensional gratings. *J. Opt. Soc. Am. A* **14**, 1592–1598 (1997).

[49] Moharam, M., Pommet, D. A., Grann, E. B. & Gaylord, T. K. Stable implementation of the rigorous coupled-wave analysis for surface-relief gratings: enhanced transmittance matrix approach. *J. Opt. Soc. Am. A*. **12**, 1077–1086 (1995).

[50] Li, L. Use of Fourier series in the analysis of discontinuous periodic structures. *J. Opt. Soc. Am. A*. **13**, 1870–1876 (1996).

[51] Henn, M.-A. et al. A maximum likelihood approach to the inverse problem of scatterometry. *Opt. Express* **20**, 12771–12786 (2012). 22714306 10.1364/OE.20.012771

[52] Dietz, N. Real-time optical characterization of thin film growth. *Mater. Sci. Eng.: B* **87**, 1–22 (2001).

[53] Theodosiou, A., Komodromos, M. & Kalli, K. Accurate and fast demodulation algorithm for multipeak fbg reflection spectra using a combination of cross correlation and Hilbert transformation. *J. Light. Technol.* **35**, 3956–3962 (2017).

[54] Chen, X., Liu, S., Zhang, C. & Jiang, H. Improved measurement accuracy in optical scatterometry using correction-based library search. *Appl. Opt.* **52**, 6726–6734 (2013). 24085171 10.1364/AO.52.006726

[55] Coulombe, S. A., Minhas, B. K., Raymond, C. J., Sohail H. Naqvi, S. & McNeil, J. R. Scatterometry measurement of sub-0.1 *μ*m linewidth gratings. *J. Vac. Sci. Technol. B* **16**, 80–87 (1998).

[56] Ruanaidh, J. J. O. & Fitzgerald, W. J. *Numerical Bayesian Methods Applied to Signal Processing* (Springer Science & Business Media, 2012).

[57] Herrero, A. F., Pflüger, M., Puls, J., Scholze, F. & Soltwisch, V. Uncertainties in the reconstruction of nanostructures in EUV scatterometry and grazing incidence small-angle X-ray scattering. *Opt. Express* **29**, 35580–35591 (2021). 34808989 10.1364/OE.430416

