# Supplementary material for: Broadband extreme ultraviolet zeroth order scatterometry for nanostructure metrology
Source: Nat Commun. 2026 May 19;17:6573. doi: 10.1038/s41467-026-73052-w (PMC13381958; doi:10.1038/s41467-026-73052-w)
Supplement: Supplementary file 1 — Supplementary Information [file 41467_2026_73052_MOESM1_ESM.pdf]

Supplementary Information for "Broadband extreme ultraviolet zeroth order scatterometry for nanostructure metrology"

Francesco Corazza[1,*], Emmanouil Kechaoglou[1], Leo Guery[1], Zhonghui Nie[1], Parikshit Phadke[1], Carl S. Lehmann[1], Roland Bliem[1,2], and Peter M. Kraus[1,3,]**

[1]Advanced Research Center for Nanolithography, Science Park 106, 1098 XG Amsterdam, The Netherlands
[2]Van der Waals–Zeeman Institute, Institute of Physics, University of Amsterdam, Science Park 904, 1098 XH Amsterdam, The Netherlands
[3]Department of Physics and Astronomy, and LaserLaB, Vrije Universiteit, De Boelelaan 1081, 1081 HV Amsterdam, The Netherlands
[*]Correspondence: f.corazza@arcnl.nl
[**]Correspondence: p.kraus@arcnl.nl

Methods

Nanostructured samples

Each scatterometry target consists of two nanoscale line gratings with a rectangular profile etched into a Silicon substrate. The samples are designed and fabricated in-house in the NanoLab Amsterdam facilities, via Electron Beam Lithography (Voyager, Raith), followed by dry etching (Cobra, Oxford Instruments). The substrate consists of Czochralski grown, Prime Grade Silicon wafers, with $\{111\}$ crystal orientation (Siegert Wafers gmbh). After the etching process and resist stripping procedures, the samples were cleaned in an Acid Piranha solution at $135°C$ for 10 minutes, followed by an O_2 plasma ashing process. The work presented in the following was carried out on a set of 36 different targets with 700 nm pitch, and a set of 4 nanostructures with pitches of 200 nm and 100 nm was fabricated to probe the applicability of the technique to more aggressively scaled geometries. The structures were made with 18 different designs, including six different groove heights between 80 and 130 nm and three different CDs. For every design we have manufactured 2 replicas. The morphology $\mathbf{p}$ of each target was characterized with both Atomic Force Microscopy (AFM) and Scanning Electron Microscopy (SEM).

After the fabrication process, the samples were characterized via X-ray Photoelectron Spectroscopy which measured a 1.2 ± 0.1 nm thick native oxide layer on the surface. The evaluation was conducted by comparing the atomic concentrations of the silicon oxide and silicon peaks in the Si-2p region within the binding energy range of $96 - 110$ eV. We note that the initial XPS measurements were performed at the stage when only the Si-2p and O-1s regions were examined for quantification of the native oxide. At that time, the presence of resist residue was not yet suspected, and the C-1s region was not analyzed in detail. The residue was identified later through tilted SEM imaging, which revealed a thin, spatially uniform film on the structured regions. This observation prompted the extra cleaning procedure and a subsequent XPS analysis explicitly targeting the C-1s region on the cleaned samples, confirming the presence of persistent carbonaceous material in the form of sp^2 and sp^3 hybridized species.

Experimental setup

The primary source is a Ti:Sapphire laser system (Solstice ACE, Spectra Physics), which offers 40 fs pulse duration and an operating repetition rate of 2 kHz. Approximately 3 W are utilized for High Harmonic Generation. The beam is focused using a 250 mm focal length lens into a 3 mm long gas cell reaching $\sim 10^{15}$ W/cm^2 peak intensity. The cell was wrapped to obtain the highest pressure difference with respect to the surrounding chamber, and pressurized with gas supplied by a mass-flow controller that ensures a stable flow and pressure. The high-harmonics generation apparatus can be switched between two configurations to allow some tunability over the XUV spectrum: one dedicated to an ordinary HHG regime, characterized by a discrete harmonics spectrum, and one where the generation conditions are tweaked to obtain a continuum-like spectrum. In the first case, the traditional phase matching conditions for HHG are met, while in the latter case the ionization rate in the gas is increased by higher field intensities. This leads to modulation of the field which reduces the overall harmonic yield in favor of creating a much more continuous spectrum. Switching between the two HHG processes is achieved by controlling the initial

diameter of the beam using a pinhole before the focusing lens, while the focusing position remains practically the same. An example of the spectral coverage is shown in Supplementary Figure 1. The switching between these two generation conditions was achieved by adjusting the focusing position inside the gas cell along the beam propagation direction and by altering the intensity of the focus beam tuning the aperture of an iris placed before the focusing lens.

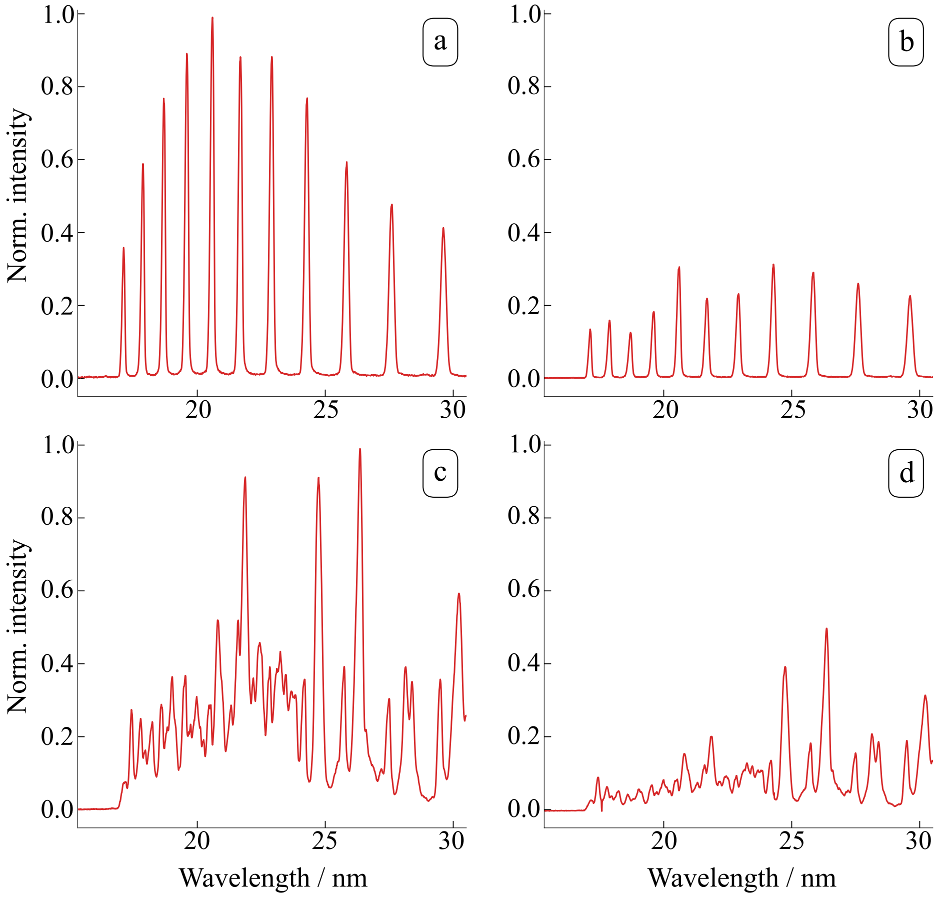

Supplementary Figure 1. XUV reflectivity spectra measured under different experimental conditions. a) Discrete spectrum, measured on a flat Silicon surface. b) Discrete spectrum, measured on a nanostructured sample. c) Nearly continuous spectrum, measured on a flat Silicon surface. d) Nearly continuous spectrum, measured on a nanostructured sample. b) and d) report the data from 0th order spectra measured on a target with 350 nm CD and 108 nm groove height, in planar diffraction conditions ($\phi = 0°$) .

Calibration of the Angle of Incidence The angle of incidence (AOI) plays a critical role in determining the diffraction efficiency. Even small deviations—as little as 0.5°, can introduce systematic errors in morphology reconstruction. To minimize this effect, we implemented a dedicated calibration routine for the incident angle θ.
A SmarAct vacuum-compatible piezo rotary stage (closed-loop operation, angular accuracy 25 $\mu°$) was used to control the AOI. Calibration was performed by aligning the sample stage to 0° AOI as follows: the attenuated IR driver laser was directed onto the sample after removal of the metallic filter and subsequently back-reflected along its original 180 cm path through the HHG gas cell. The 0° AOI position was defined as the stage setting that maximized the amount of light back-coupled into the gas cell. This method exploits the narrow acceptance angle of the toroidal mirror and the small aperture of the gas cell to provide a sensitive alignment condition. In the experimental configuration, the 78° AOI was set and measured using the closed-loop controller. Reproducibility tests yielded a standard deviation of 0.002°.

Data Processing

For quasi-continuous XUV spectra, the relative reflectivity was directly derived by dividing the measured zeroth-order spectrum by that obtained from a pristine silicon reference surface. A narrow Gaussian low-pass filter was applied in the Fourier space to minimize artifacts caused by rapid signal oscillations while preserving the overall signal integrity. It is important to note that the beyond phase-matching regime usually presents spectral fluctuations that are significantly larger than those of the optimal HHG

regime. For this reason, we chose to discuss and show our morphology reconstruction results for the optimal HHG regime, while we only use data acquired with a nearly continuous illumination spectrum to choose the most fitting interpolation routine that best describes the signal measured with a continuous illumination spectrum. After exploring different fitting methods, such as polynomial and sums of trigonometric functions, we concluded that the most suitable fitting procedure for the hereby observed relative reflectivity signal is the piecewise polynomial interpolation, also known as the spline interpolation routine, as shown in Supplementary Figure 2. It emerges that the curve fitted to the measured relative reflectivity with discrete HHG correctly maps the signal measured with nearly continuous spectrum within the $1-\sigma$ uncertainty interval.

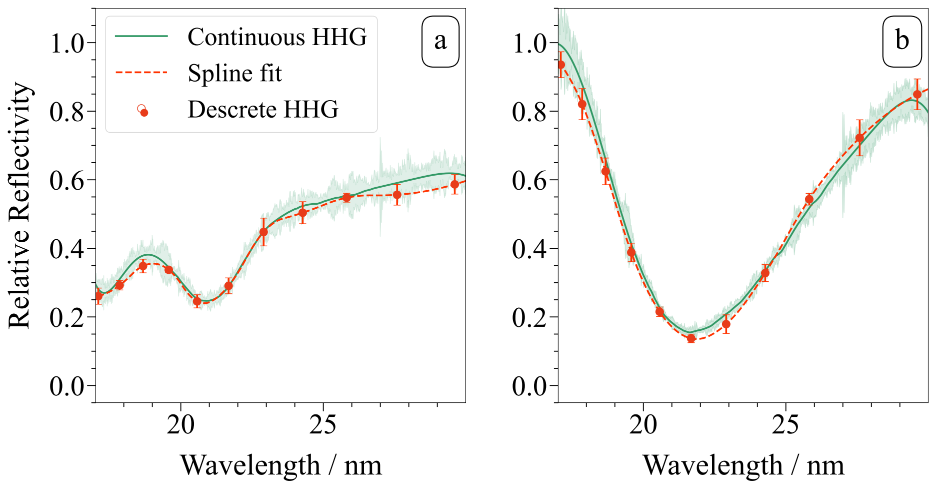

Supplementary Figure 2. Comparison of the measured relative reflectivities probed with XUV light generated in both discrete and continuum-like HHG regime. a) Planar diffraction configuration ($\phi = 0°$). b) Conical diffraction configuration ($\phi = 90°$).

Results

Supplementary Tables 1 and 2 provide a complete overview of the morphology reconstruction results of the nanostructures with 100 nm pitch. Similarly, Supplementary Tables 3 and 4 report the results for the metrology targets with a pitch of 200 nm. Supplementary Tables 5 and 6 provide a complete overview of the reconstruction results obtained with the 0^{th}order XUV scatterometry method for all 36 metrology targets investigated in this work. For each target, the reconstructed geometric parameters are reported together with the corresponding independently measured reference values (AFM for groove height and SEM for critical dimension). In addition, the tables include the reconstruction uncertainty derived from the χ^2 confidence analysis, as well as the reconstruction bias and the normalized error (z-score).

The data supporting Figure 8-a, labeled as 1, 2, 3, 4, 5 and 6 concern targets 1, 7, 17, 18, 25 and 33, respectively. The data supporting Figure 8-b, labeled as 7, 8 and 9 concern targets 7, 10 and 12, respectively.

After carefully inspecting the sample and finding local damage to the nanostructure, Target 36 was considered an outlier and was not included in the uncertainty validation procedure for both GH and CD.

Supplementary Table 1. 100 nm pitch metrology targets. Groove Height accuracy results.

Target	AFM Ref. (nm)	LSR (nm)	CI χ^2 (nm)	Bias	σ_c	$z-score$
Target C	68.20 ± 0.40	69.81	$-0.44/+0.40$	1.61	0.58	2.77
Target D	68.20 ± 0.40	70.58	$-0.75/+0.75$	2.38	1.38	2.03

Supplementary Table 2. 100 nm pitch metrology targets. Critical Dimension accuracy results.

Target	SEM Ref. (nm)	LSR (nm)	CI χ^2 (nm)	Bias	σ_c	$z-score$
Target C	50.00 ± 2.50	46.68	$-3.31/+2.35$	3.32	4.72	0.70
Target D	50.00 ± 2.50	56.59	$-4.59/+3.65$	6.59	6.34	1.03

Supplementary Table 3. 200 nm pitch metrology targets. Groove Height accuracy results.

Target	AFM Ref. (nm)	LSR (nm)	CI χ^2 (nm)	Bias	σ_c	$z-score$
Target A	68.20 ± 0.40	67.18	$-1.23/+1.15$	-1.02	1.25	-0.81
Target B	68.20 ± 0.40	67.80	$-0.66/+0.62$	-0.40	0.75	-0.53

Supplementary Table 4. 200 nm pitch metrology targets. Critical Dimension accuracy results.

Target	SEM Ref. (nm)	LSR (nm)	CI χ^2 (nm)	Bias	σ_c	$z-score$
Target A	80.00 ± 2.50	84.66	$-6.63/+6.80$	3.30	6.94	0.47
Target B	80.00 ± 2.50	70.57	$-6.51/+7.42$	-9.43	7.19	-1.31

Supplementary Table 5. 700 nm pitch metrology targets. Groove Height (GH) accuracy results.

Target	AFM Ref. (nm)	LSR (nm)	CI χ^2 (nm)	Bias	σ_c	$z-score$
Target 1	80.00 ± 0.30	80.63	$-0.70/+0.70$	0.63	0.76	0.83
Target 2	80.00 ± 0.30	79.93	$-1.00/+2.70$	-0.07	1.88	-0.04
Target 3	80.00 ± 0.30	80.03	$-0.60/+0.80$	0.03	0.76	0.04
Target 4	80.00 ± 0.30	80.63	$-0.70/+0.70$	0.63	0.76	0.83
Target 5	80.00 ± 0.30	80.03	$-0.90/+1.40$	0.03	1.19	0.03
Target 6	80.00 ± 0.30	82.03	$-1.00/+0.90$	2.03	1.00	2.04
Target 7	92.00 ± 0.50	92.44	$-1.00/+0.90$	0.44	1.07	0.41
Target 8	92.00 ± 0.50	92.54	$-1.00/+0.70$	0.54	0.99	0.55
Target 9	92.00 ± 0.50	91.34	$-1.10/+0.90$	-0.66	1.12	-0.59
Target 10	92.00 ± 0.50	91.04	$-0.80/+1.10$	-0.96	1.07	-0.89
Target 11	92.00 ± 0.50	91.34	$-1.00/+0.90$	-0.66	1.07	-0.61
Target 12	92.00 ± 0.50	89.34	$-1.30/+1.10$	-2.66	1.30	-2.04
Target 13	104.00 ± 0.50	106.66	$-0.90/+1.00$	2.66	1.07	2.47
Target 14	104.00 ± 0.50	104.75	$-0.80/+2.60$	0.75	1.77	0.43
Target 15	104.00 ± 0.50	104.95	$-0.70/+2.00$	0.95	1.44	0.66
Target 16	104.00 ± 0.50	103.75	$-0.90/+1.00$	-0.25	1.07	-0.23
Target 17	104.00 ± 0.50	103.45	$-1.00/+1.00$	-0.55	1.12	-0.49
Target 18	108.00 ± 0.30	108.36	$-1.00/+1.00$	0.36	1.04	0.34
Target 19	108.00 ± 0.30	107.96	$-0.80/+0.80$	-0.04	0.86	-0.05
Target 20	108.00 ± 0.30	108.16	$-0.80/+1.00$	0.16	0.95	0.17
Target 21	108.00 ± 0.30	108.06	$-1.00/+1.00$	0.06	1.04	0.06
Target 22	108.00 ± 0.30	108.96	$-0.70/+0.60$	0.96	0.72	1.34
Target 23	108.00 ± 0.30	108.26	$-0.80/+1.30$	0.26	1.09	0.24
Target 24	116.00 ± 0.20	117.67	$-1.20/+0.70$	1.67	0.97	1.72
Target 25	116.00 ± 0.20	117.37	$-0.90/+0.70$	1.37	0.83	1.66
Target 26	116.00 ± 0.20	115.97	$-1.00/+1.00$	-0.03	1.02	-0.03
Target 27	116.00 ± 0.20	115.57	$-0.80/+1.40$	-0.43	1.12	-0.39
Target 28	116.00 ± 0.20	116.97	$-2.30/+0.70$	0.97	1.51	0.64
Target 29	116.00 ± 0.20	117.07	$-0.70/+0.70$	1.07	0.73	1.46
Target 30	122.00 ± 0.50	122.77	$-1.00/+1.00$	0.77	1.12	0.69
Target 31	122.00 ± 0.50	122.77	$-0.90/+1.10$	0.77	1.12	0.69
Target 32	122.00 ± 0.50	122.27	$-0.80/+2.30$	0.27	1.63	0.17
Target 33	122.00 ± 0.50	122.67	$-1.10/+1.30$	0.67	1.30	0.52
Target 34	122.00 ± 0.50	124.87	$-0.80/+0.90$	2.87	0.99	2.91
Target 35	122.00 ± 0.50	120.87	$-0.70/+0.90$	-1.13	0.94	-1.20
Target 36	104.00 ± 0.50	56.31	$-6.31/+55.26$	-47.69	30.78	-1.55

Supplementary Table 6. 700 nm pitch metrology targets. Critical Dimension (CD) accuracy results.

Target	SEM Ref. (nm)	LSR (nm)	CI χ^2 (nm)	Bias	σ_c	$z-score$
Target 1	290.00 ± 2.50	295.88	$-6.80/+7.01$	-5.88	7.12	-0.83
Target 2	290.00 ± 2.50	296.31	$-8.71/+9.77$	-6.31	9.40	-0.67
Target 3	340.00 ± 2.00	348.32	$-9.34/+9.98$	-8.32	9.76	-0.85
Target 4	340.00 ± 2.00	345.99	$-9.34/+10.40$	-5.99	9.97	-0.60
Target 5	390.00 ± 2.30	399.70	$-13.17/+13.80$	-9.70	13.58	-0.71
Target 6	390.00 ± 2.30	408.41	$-44.80/+96.19$	-18.41	70.52	-0.26
Target 7	290.00 ± 2.50	291.64	$-4.88/+4.88$	-1.64	5.19	-0.31
Target 8	290.00 ± 2.50	298.22	$-5.95/+5.95$	-8.22	6.20	-1.32
Target 9	340.00 ± 2.00	349.60	$-9.77/+9.77$	-9.60	9.87	-0.97
Target 10	340.00 ± 2.00	347.47	$-7.01/+7.22$	-7.47	7.25	-1.03
Target 11	390.00 ± 2.30	400.34	$-14.86/+14.23$	-10.34	14.64	-0.71
Target 12	390.00 ± 2.30	388.24	$-13.38/+14.86$	1.76	14.21	0.12
Target 13	290.00 ± 2.50	296.09	$-7.22/+7.64$	-6.09	7.64	-0.80
Target 14	290.00 ± 2.50	320.94	$-11.47/+11.47$	-30.94	11.60	-2.67
Target 15	340.00 ± 2.00	332.82	$-17.20/+16.78$	7.18	17.05	0.42
Target 16	340.00 ± 2.00	340.25	$-9.56/+10.19$	-0.25	9.97	-0.03
Target 17	390.00 ± 2.30	376.77	$-21.87/+24.84$	13.23	23.41	0.56
Target 18	390.00 ± 2.30	376.56	$-16.56/+18.69$	13.44	17.70	0.76
Target 19	290.00 ± 2.50	254.47	$-10.83/+10.83$	35.53	10.97	3.24
Target 20	340.00 ± 2.00	342.17	$-7.64/+7.64$	-2.17	7.77	-0.28
Target 21	340.00 ± 2.00	343.65	$-8.07/+8.28$	-3.65	8.30	-0.44
Target 22	390.00 ± 2.30	388.24	$-10.40/+11.04$	1.76	10.85	0.16
Target 23	390.00 ± 2.30	384.84	$-11.89/+12.53$	5.16	12.32	0.42
Target 24	290.00 ± 2.50	289.09	$-5.73/+5.95$	0.91	6.10	0.15
Target 25	290.00 ± 2.50	289.51	$-4.88/+5.31$	0.49	5.39	0.09
Target 26	340.00 ± 2.00	339.62	$-7.01/+7.43$	0.38	7.36	0.05
Target 27	340.00 ± 2.00	342.38	$-7.86/+7.86$	-2.38	7.98	-0.30
Target 28	390.00 ± 2.30	375.71	$-12.53/+13.80$	14.29	13.27	1.08
Target 29	390.00 ± 2.30	385.90	$-11.68/+13.17$	4.10	12.53	0.33
Target 30	290.00 ± 2.50	283.78	$-5.52/+5.52$	6.22	5.80	1.07
Target 31	290.00 ± 2.50	283.35	$-5.10/+5.52$	6.65	5.60	1.19
Target 32	340.00 ± 2.00	333.46	$-8.07/+8.92$	6.54	8.61	0.76
Target 33	340.00 ± 2.00	348.32	$-7.43/+7.86$	-8.32	7.77	-1.07
Target 34	390.00 ± 2.30	378.47	$-21.23/+29.52$	11.53	25.43	0.45
Target 35	390.00 ± 2.30	415.21	$-8.28/+8.71$	-25.21	8.65	-2.91
Target 36	290.00 ± 2.50	257.02	$-9.13/+9.34$	32.98	9.40	3.51
