# Supplementary material for: Broadband extreme ultraviolet zeroth order scatterometry for nanostructure metrology
Source: Nat Commun. 2026 May 19;17:6573. doi: 10.1038/s41467-026-73052-w (PMC13381958; doi:10.1038/s41467-026-73052-w)
Supplement: Supplementary file 2 — Transparent Peer Review file [file 41467_2026_73052_MOESM2_ESM.pdf]

nature portfolio

Peer Review File

Broadband extreme ultraviolet zeroth order scatterometry for nanostructure metrology

Corresponding Author: Dr Peter Kraus

This file contains all reviewer reports in order by version, followed by all author rebuttals in order by version.

Attachments originally included by the reviewers as part of their assessment can be found at the end of this file.

Version 0:

Reviewer comments:

Reviewer #1

(Remarks to the Author)
Please find attached review document in pdf-format. Additionally highlighted manuscript and supplementary information files attached.

Reviewer #2

(Remarks to the Author)
I co-reviewed this manuscript with one of the reviewers who provided the listed reports. This is part of the Nature Communications initiative to facilitate training in peer review and to provide appropriate recognition for Early Career Researchers who co-review manuscripts.

Reviewer #3

(Remarks to the Author)
The author presents their scatterometry work using a broadband extreme ultraviolet source for establishing metrology on every shrinking critical dimension in nanofabrication.
The article is divided into two main sections. A rather small section that describes the light source and a much longer section that describes how the scatterometry samples and analysis. The samples have rather dimensions relative to the aim of providing a metrology solution on critical dimension for state of the are nanofabrication. It is possible to chararterize the presented samples without EUV light.
The presented scatterometry analysis method is well known and has been reported in many articles before.
The main significance of the article is the light source. Working in the EUV regime using a tabletop source higher harmonic generation from a gass cell is a significant step forward from synchroton source and laser-produced plasma source at 13.5-nanometer. I therfore suggest that the focus of the article should be shifted more towards the light source.

The article is in general well written and clearly explained.

The authors claims that the 0'th order is seldom used (forgotten) in scatterometry. This is not correct it is the most used order in spectroscopic scatterometry. This statement should be corrected.

I cannot recommend the article for nature communications in its present form. However, I would encourage the author to resubmit a new manuscript with more focus on the source and with investigation on samples with smaller dimensions.

Reviewer #4

(Remarks to the Author)
This paper explores a particular modality of EUV reflectometry/scatterometry for use in measuring critical dimensions and

groove depths of gratings in a semiconductor setting. The approach is relatively simple, and relies on matching carefully considered measurements to a pre-simulated library of diffraction efficiencies computed using RCWA. The results are surprisingly accurate considering that EUV scattering is usually sensitive to any/all aspects of a sample (intended or not); in part, this indicates a very high quality of sample fabrication. In addition, the paper is well-written with clear figures and proper technical information. I recommend it for publication.

Minor suggestions
- In Section 2.2, the authors mention the angle of incidence as 78deg. Much of their audience will likely come from the fields of EUV and soft x-rays, so they could consider specifying that this is 78deg from normal incidence.
- In Figure 3 the authors could consider adding the labels "Planar Diffraction" and "Conical Diffraction" above plots (a) and (b), respectively.
- supp figure 2 caption is missing a closing parenthesis

Version 1:

Reviewer comments:

Reviewer #1

(Remarks to the Author)
We thank the authors for their thorough revision of their manuscript and for carefully addressing our comments and questions. Especially, the revised discussion of uncertainties is a great addition and strengthens the impact of their paper.

In a side note we want to address that one referenced paper, Lohr et al. (Ref 21), still appears to be misinterpreted in the given response letter (RR1-11/RR3-2). In the paper two metrology setups are compared of which one in fact does already apply a broadband zeroth order scatterometry approach without a monochromator, see also the referenced paper by Bahrenberg et al. (Ref. 22). Here, not a HHG source but a DPP source is used, so the novelty of a broadband zeroth order EUV scatterometry setup with an HHG source still applies.

We are pleased to recommend the acceptance of the paper without further revision.

Reviewer #2

(Remarks to the Author)
I co-reviewed this manuscript with one of the reviewers who provided the listed reports. This is part of the Nature Communications initiative to facilitate training in peer review and to provide appropriate recognition for Early Career Researchers who co-review manuscripts.

Reviewer #3

(Remarks to the Author)
The author has carefully adressed all of the reviewers questions from the first review, resulting in a much improved version of the article.

It is a promising technology for inline quality control of wafer prodution once the large spot size problem has been resolved.

I have no problem in recomending the updated article for publication in Nature.

Reviewer #4

(Remarks to the Author)
All of my comments have been addressed sufficiently.

Open Access This Peer Review File is licensed under a Creative Commons Attribution 4.0 International License, which permits use, sharing, adaptation, distribution and reproduction in any medium or format, as long as you give appropriate credit to the original author(s) and the source, provide a link to the Creative Commons license, and indicate if changes were made.
In cases where reviewers are anonymous, credit should be given to 'Anonymous Referee' and the source.
The images or other third party material in this Peer Review File are included in the article's Creative Commons license, unless indicated otherwise in a credit line to the material. If material is not included in the article's Creative Commons license and your intended use is not permitted by statutory regulation or exceeds the permitted use, you will need to obtain permission directly from the copyright holder.
To view a copy of this license, visit https://creativecommons.org/licenses/by/4.0/

Nat. Communications

Ref.: **Response letter to referee reports**
Manuscript NCOMMS-25-58161

Amsterdam, January 29th, 2026

E-mail: kraus@arcnl.nl

Dear Referees:

We express our gratitude to the referees for their expert feedback and valuable suggestions on the manuscript titled: "Broadband extreme ultraviolet zero-order Scatterometry for nanostructure metrology" by Corazza et al. We took into consideration all the referee's comments and addressed them in the manuscript. In this response letter we provide a point-by-point response to the comments received from the reviewers. A short summary of changes is attached as well. The referees' comments are indicated in **blue**, our responses are in **black**, and any changes are highlighted in **red**.

Report of Referee #1 and #2:

#1 Please find attached review document in pdf-format. Additionally highlighted manuscript and supplementary information files attached.

#2 I co-reviewed this manuscript with one of the reviewers who provided the listed reports. This is part of the Nature Communications initiative to facilitate training in peer review and to provide appropriate recognition for Early Career Researchers who co-review manuscripts.

Authors' reply: we thank you for the detailed report you have provided. It offered us the opportunity to improve the manuscript and to dive deeper into experimental details. With the goal of structuring our answers as much as possible, we took the freedom to number the comments received in the PDF files.

RR1 - 1 Good introduction to a new setup capable of EUV scatterometry with well described setup, process and results. Good use of illustrations and understandable plots.

Authors reply: We greatly appreciate your positive comment. Thank you.

Change: None.

RR1 - 2 Generally, the presented metrology method is not new, and with the presented setup and reconstruction algorithm it is less powerful than already existing setups, still the application of a "no-calibration" method is interesting. But it should be made clearer from the beginning, that a reference measurement (of a non-structured) sample is required, which kind of negates the "no-calibration" approach.

Authors reply: Thank you for the comment. First of all, we would like to point out that our approach is new in the implementation of 0th order scatterometry in an HHG setup using broadband illumination, please see answer to comment RR1 – 11. We acknowledge that, for certain parameters, the sensitivity of our current setup and reconstruction can be lower than that of some established, specialized instruments; however, our approach is fully tabletop, offers (in principle) high throughput, and is robust.
Regarding the "nocalibration" description, we agree that a **reference measurement** is required. In our protocol, the reference is taken on an **unstructured area of the same substrate**, adjacent to the metrology target. This step is straightforward in many settings, both industrial and academic. We would like to state that the need of a reference measurement does not negate the "no-calibration approach". We avoid any **absolute** calibration of the system throughput (toroidal mirror, metallic filters, dispersive grating, CCD) and of the HHG source spectrum, which would otherwise require a dedicated apparatus. Furthermore, all the components of such a setup should be regularly calibrated since aging and the continuous exposure to XUV light (and IR light for metallic filters) significantly alter the instrumental response function of the system. This would be impractical for any in-line metrology application.
Our approach only requires an additional spectral measurement that does not require precise absolute reflectivity calibration of any optical component. Moreover, there is no need for additional apparatus to acquire the HHG spectrum coming from the source.

We value the suggestion of making the advantages of the no-calibration approach with reference measurement more explicit.

Change: We have decided to change the sentence "This approach eliminates the necessity of extracting any absolute reflectivity terms, which are often challenging due to the need for precise instrumentation calibration and XUV reference measurements." in the main text with the more detail period: "This approach avoids extracting absolute reflectivity terms by normalizing each spectrum to a local reference acquired on an unstructured area of the same substrate, adjacent to the target. Working with relative reflectivity obviates the absolute calibration of the setup components, such as toroidal mirror, metallic filters, flat-field grating, CCD response, and the HHG source spectrum—quantities that drift with XUV exposure and aging—while preserving robustness and high throughput.".

RR1 - 3 The uncertainties derived for morphology reconstruction are, in our view, incorrect, and should be revised. They are given as the database increment step, which is mainly based on the fact that the averaging of measurements between different samples (which should be similar but have intrinsic variations e.g. due to fabrication) yields smaller uncertainties, instead of basing the uncertainty on the intrinsic uncertainty of the LSR. Additionally, systematic errors during the experiment (e.g. on incidence angle, azimuthal angle, etc.) seem to be neglected, which in our experience can have a major influence on reconstruction accuracy (small change in incidence angle e.g. can lead to systematic shift of diffraction minima & maxima), even within the "no-calibration" approach.

Authors reply: We thank the referees for the comments. It proved to be very valuable for us, and a good starting point to review our approach to uncertainties in more detail.

In the original submission, reconstruction uncertainties were effectively tied to the parameter discretization step of the RCWA database. We agree with the referee that this is not the most statistically rigorous estimate of reconstruction uncertainty. In the revision, we now determine uncertainties using a χ^2-based weighted least-squares framework, where for each reconstruction the confidence interval is extracted from the χ^2 landscape via the standard criterion $\Delta\chi^2 = 1$ (68% confidence for one fitted parameter). This yields confidence bounds $(p_{\text{best}} - \Delta p^{-}, p_{\text{best}} + \Delta p^{+})$, which we directly report as the *intrinsic reconstruction uncertainty* for both GH and CD.
We want to clearly state that this procedure does not rely on averaging different targets and does not scale with database step size. Instead, it is determined by the sensitivity of the forward model and by the noise/variance of the measured spectrum.

We further agree that systematic experimental errors can introduce structured deviations and systematic shifts of diffraction features. Since these effects are not captured by repeated-measurement scatter alone, we now include an additional systematic uncertainty contribution in the χ^2 weighting term. Concretely, we have decided to add to the experimental variance a term, structured as follows: $\sigma^2_{\lambda,\text{p}}(sys) = a \cdot F_{\lambda,\text{p}} + b$, as inspired by literature literature.

We thank the referee for encouraging us to revise and strengthen the uncertainty framework. This revision motivated a substantial update of the manuscript figures: in particular, we replaced the previous Figures 6 and 7, which visualized the normalized Euclidean distances used during the reconstruction process, with the new Figure 8. The new figure provides a clearer and more intuitive representation of the reconstruction results and explicitly supports

the assessment of reconstruction accuracy and the validation of the χ^2-derived confidence intervals.

We note that the referee's concerns about incidence and azimuth angle calibration are also addressed in the revised manuscript discussion and further elaborated in the response to RR1–20.

Change: The LSR routine was updated, and the reconstructions where repeated from scratch, and the text of the main article was expanded to the following: "

$$\chi^2_{\lambda,\mathbf{p}} = \frac{[\delta R_\lambda - \mathscr{F}_{\lambda,\mathbf{p}}]^2}{\sigma^2_{\lambda,\mathbf{p}}}$$

$$\sigma^2_{\lambda,\mathbf{p}} = \sigma^2_{\lambda\,(\mathrm{exp})} + \sigma^2_{\lambda,\mathbf{p}\,(sys)}$$

where $F_{\lambda,\mathrm{p}}$, p represents the simulated relative reflectivity for the p morphology and $\sigma^2_{\lambda,\mathrm{p}}$ is the variance. The latter is composed of two terms: the first one accounts for the measured uncertainty of the experiments, and the second includes linearly dependent systematic errors [45]. In this case we used $\sigma^2_{\lambda,\mathrm{p}}(sys) = a \cdot F_{\lambda,\mathrm{p}} + b$."

For the rest of the changes concerning the uncertainty validation and reconstruction accuracy please look directly at the revised document. The changes involve the second half of the Results section and the first half of the Discussion section.

RR1 - 4 Generally, the used sample parameters seem to be no good match for the used wavelength range which could be an explanation why data shows low sensitivity to CD.

1. In my opinion, the diffraction of 10-30 nm radiation at 700 nm is very low.
2. On the other hand, the visible sensitivity to the line height might mainly come form "thin-film" interference between beams reflected of the upper and lower surfaces of the grating. This issue should be addressed in the paper.
3. An additional study on gratings with smaller pitches and the expected sensitivity to CD and line height could show if sensitivity might improve for other grating parameters.

Authors reply: We thank the referee for this comment. Our reply is structured in three points below. We are convinced that the limited CD sensitivity is connected to the short wavelength and relatively large structural parameters. The explanation follows in points below.

1. The statement that "the diffraction of 10–30 nm radiation at 700 nm is very low" is somewhat vague, so we interpret it in terms of the diffraction efficiencies of the 0th and first orders. Within this framework, we believe that the statement that the diffraction efficiency is "very low" is not correct. We have therefore added plots in this wavelength range, computed with our RCWA simulator, for two different structures with 700 nm and 100 nm pitches, both with 50% duty cycle and 100 nm groove height (see new Figures a and b below). In these plots we report the diffraction efficiencies, which by construction include the substrate reflectivity.
The data behind the 700 nm plot were calculated at the same incidence angle used in the submitted manuscript, 78°, while the 100 nm-pitch plot was calculated for a 70° incidence

angle. This choice is dictated by the sensitivity in the planar diffraction configuration, where there is a trade-off between pitch size and incidence angle to maintain a comparable level of sensitivity. In this context, we note that the reflectivity of Si at 70° is nearly one order of magnitude lower than at 78°.

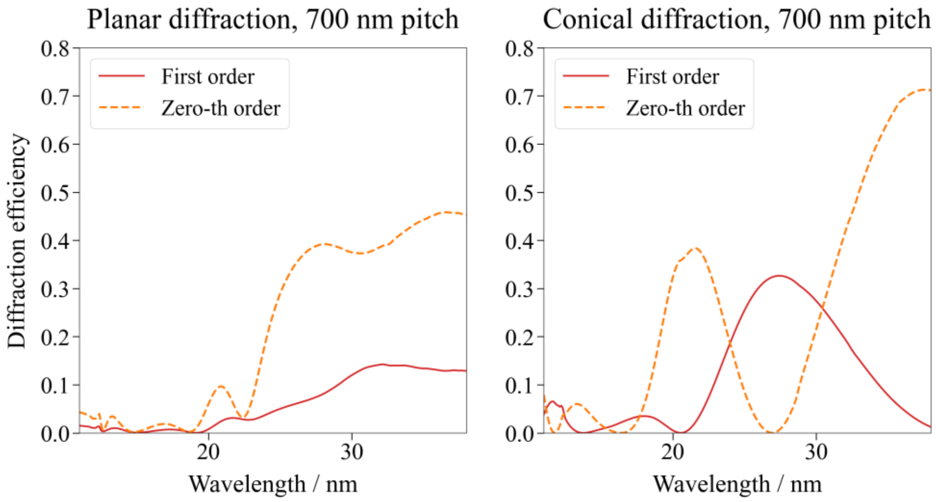

Figure a – Diffraction efficiency from a nanostructure 700 nm pitch and 50 duty cycle, in both planar and conical diffraction. Simulation performed with an incidence angle of 70°.

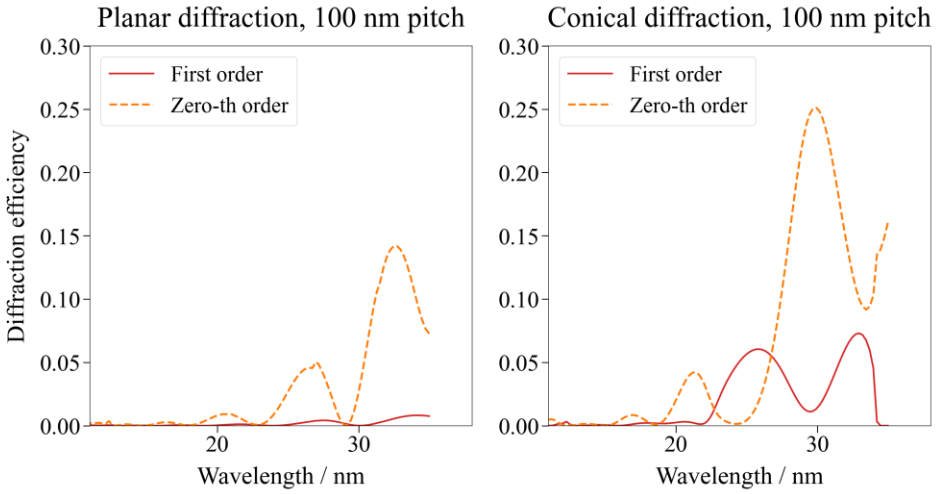

Figure b – Diffraction efficiency from a nanostructure 100 nm pitch and 50 duty cycle, in both planar and conical diffraction. Simulation performed with an incidence angle of 70°.

From these two plots it is apparent that the diffraction efficiencies of both the zeroth and first orders are consistent and relatively large, and therefore not “very low” in the above sense.

2. The “thin-film” interference effect mentioned in the comment is indeed an intrinsic and crucial component of diffraction from binary gratings in our geometry. In our RCWA modeling, the interference between fields reflected from the top and bottom surfaces of the grating lines is fully included and is responsible for the pronounced spectral structure of the zeroth order. What we are observing is therefore genuinely grating diffraction, as is

further evidenced by the appearance of higher diffraction orders in the conical diffraction regime (see the above plots).
To address the referee's concern, we have added a short clarification in the manuscript explicitly.

3. We thank the referee for this suggestion. In response, we have extended our experimental study to include additional nanostructured gratings with smaller pitches of 200 nm and 100 nm.
Measurements were performed on four new targets (two per pitch) using the same broadband zeroth-order XUV scatterometry approach and reconstruction framework as for the 700 nm-pitch dataset.
The results show that the relative reflectivity spectra retain clear morphology-dependent spectral features at reduced pitches and remain in good agreement with RCWA simulations. Quantitative morphology reconstruction confirms nanometer-level accuracy for groove height and few-nanometer to ∼10 nm-level accuracy for critical dimension, comparable to the performance obtained for the 700 nm-pitch structures.
These results demonstrate that the sensitivity of the zeroth-order signal is preserved for substantially smaller pitches. This confirms the applicability of the technique beyond the original parameter set.
The measurements of the smallest pitches we are showing are now limited by the sample fabrication tools (e-beam) that are at our disposal in our clean room facility.

Change: Two different changes were implemented:

1. The content of the paper has been made more explicit as suggested by the referee: "…under conical diffraction conditions the diffraction efficiency profiles are largely independent of CD, as shown in the 4(d), while plot 4(b) highlights a strong dependence on groove height, making this configuration ideal for groove height reconstruction. Diffraction inherently includes the phase accumulated over the line height and the interference of fields within the grating profile while variations in CD act only as a relatively small perturbation on this dominant response. This effect can be exploited to disentangle the CD and groove height contributions on the 0th order spectrum."
2. The text in the main article was expanded with the following additions. Methods: "In addition to the 700 nm pitch targets, a set of 4 nanostructures with pitches of 200 nm and 100 nm was fabricated to probe the applicability of the technique to more aggressively scaled geometries. For each pitch, two independent targets were produced on the same Silicon substrate. We followed the same design and fabrication methodology as for the 700nm pitch structures, with CDs corresponding to 50% duty-cycle."
Results: "Furthermore, we performed additional measurements on gratings with pitches of 200nm and 100nm to evaluate the reconstruction capabilities of this approach at reduced feature sizes. Fig. 7 shows the relative reflectivity spectra measured on representative targets for each pitch, acquired in both planar and conical diffraction geometries and compared to RCWA simulations. These measurements were collected with an incidence angle of 70◦ , as the sensitivity to transverse features is relatively low at 78◦ for these feature sizes. Despite the substantially smaller pitch, the measured spectra exhibit clear and reproducible wavelength-dependent modulations across the probed spectral range. Detailed numbers can be found in the Supplementary Information.

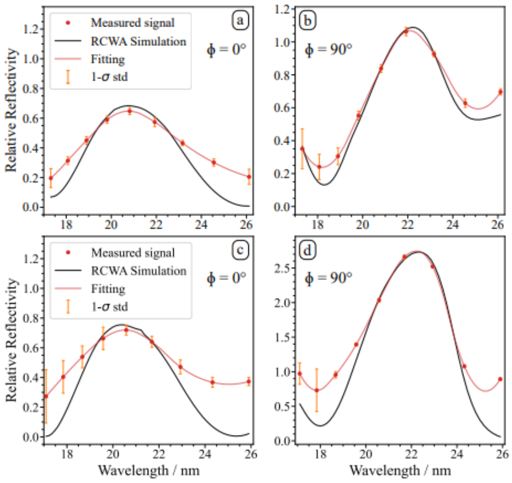

Figure 7. Relative reflectivity measurements for 100 and 200 nm pitch targets. Relative reflectivity measurements for 2 different metrology targets, plotted together with the simulated curves for the two different diffraction regimes. a) and b) report the relative reflectivity measured, respectively, in planar and conical diffraction, for a metrology target with 200 nm pitch and 100 nm nm CD. c) and d) report the relative reflectivity measured, respectively, in planar and conical diffraction, for a metrology target with 100 nm pitch and 50 nm CD. Both targets have a GH of 68 nm."

Discussion: "Morphology reconstruction was also performed for the four small-pitch targets using the same library-based least-squares regression. For the 200 nm pitch targets, the reconstructed groove heights agree with the reference AFM values within 1.1 nm, with combined uncertainties below 1.3 nm. The corresponding critical dimensions are reconstructed within 5−10 nm of the SEM reference values, consistent with the performance observed for the 700 nm pitch dataset. For the 100 nm pitch targets, groove height reconstruction remains accurate at the nanometer level, with deviations from the reference values around 2 nm. The reconstruction of the critical dimension yields 3−7 nm (absolute) bias values, with uncertainty intervals that remain statistically consistent with the observed deviations."

RR1 - 5 The amount of measured datapoints used for fitting with the database is a bit low. To be honest, seeing figure 5, fitting a curve to the 11 datapoints that is shifted by one or two nm (in wavelength) wouldn't be so bad either, which would have a major influence on the reconstructed morphology parameters.

Authors reply: Thank you for the comment. There are substantially more than 11 datapoints in the fit, as we also generate and use extreme ultraviolet with a broadband spectrum (now Fig. 3 in manuscript, but it has already been used previously in the reconstruction). The fitting occurs through a routine for the minimization of the Euclidean distances. The fitting procedure was validated by comparing the data acquired with the 'discrete' XUV illumination spectrum and the 'continuous' illumination spectrum, as illustrated in the Supplementary methods in the "Data Processing" section and Supp Figure 3. Here the wavelength mismatch is much smaller than 1 nm.

Change: We have added the following image to the main body of the article, followed by its caption:

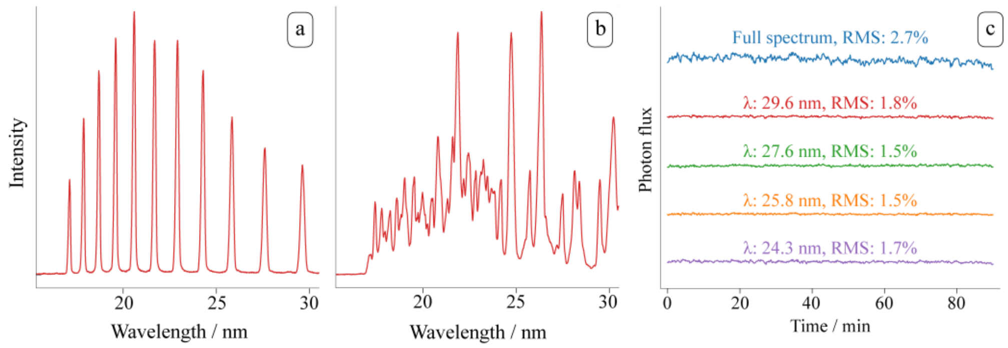

Figure 3. HHG illumination spectrum. a) XUV illumination spectrum obtained in optimal generation conditions, characterized by a discrete spectrum. b) XUV illumination with a continuum-like spectrum. c) Source stability traces for a 90 minutes long measurement in the discrete-spectrum generation regime with a 2s exposure time. We show the stability trace for four different harmonics, and also the stability trace of the total HHG spectrum.

RR1 - 6 Due to the "no-calibration" method the current data fitting mainly considers the position of interference maxima and minima and is incapable of fitting the amplitude, which already makes CD reconstruction harder than line height reconstruction.
To enlarge the amount of morphology parameters in future research, an extensive study should be done, to determine if the presented method has any sensitivity to additional morphology parameters, and where it will fall short. This should also be made clearer in the Conclusion.

Authors reply: Thanks for the comment. The "Features tracking" approach presented in the article is based on the identification of maxima and minima, and we concur on its limitations in terms of morphology reconstruction capabilities. However, we believe that the first statement of this comment is based on a misunderstanding about the reconstruction (fitting) procedure. The features tracking approach is not used as reconstruction approach, but it is only used as additional (auxiliary) step for the LSR-based reconstruction.
As explained in the paper, features tracking is used to "narrow down library items to a subset that closely matches the experimental data" to simplify the library search problem. The actual reconstruction happens by means of the LSR approach, based on the comparison of the measured data with the remaining candidate in the simulated database. We understand that it could be made clearer and we are taking action for it.
We thank the referees for the suggestion of a more complete morphology reconstruction. The follow-up of this work will be focused on the sensitivity of 0th order scatterometry to a larger number of parameters, and it will mainly focus on the use of Simulation Base Inference for morphology reconstruction instead of LRS. This approach is based on Maximum Likelihood estimation theory and exploits neural networks to linearize the inverse problem. However, this approach goes far beyond the scope of this paper, which aims to communicate the usefulness of broadband 0th order scatterometry based on a table-top HHG source. We value the suggestions and make it more explicit in the conclusions.

Change: In the Morphology reconstruction section, the Features tracking subsection was moved before the Least Square regression subsection.

A paragraph in the conclusive remarks was modified as follows: “Looking forward, the next natural step is the extension of the morphology reconstruction algorithm to automatic differentiation in the Simulator-Based Inference framework, with the goal of investigating more complex samples with a large parameters space to explore the sensitivity limits of this technique.”

RR1 - 7 “Most industrial metrology is carried out on periodic structures to increase diffraction efficiencies and lower costs.” Sentence a bit hard to read. Maybe it combines several aspects: Most industrial metrology uses periodic structure fields for quality and process control. The diffraction efficiency can be used to determine the test structure geometry that is indirectly linked to the fabrication process of the device structures.

Authors reply: Thank you for the suggestion. We agree and we have decided to modify the abstract.

Change: The sentence was changed to ease the article read, the abstract was changed as follows: ” The continuous shrinkage of the Critical Dimension in nanofabrication demands short-wavelength nanometrology. Most industrial metrology uses periodic structures for quality and process control, exploiting diffraction to probe the fabrication quality of actual device structures with a low cost. We introduce a table-top High-Harmonic-Generation extreme-ultraviolet scatterometry with broadband illumination. Our method exploits the spectrally resolved 0th diffraction order of the extreme-ultraviolet light, which, while lacking spatial-encoded information, carries valuable spectral information and offers high diffraction efficiency. The use of relative reflectivity removes the need for absolute calibration, and Rigorous Coupled-Wave Analysis simulations underpin a library-based reconstruction approach, yielding single-nanometer accuracy for groove height and 10 nm accuracy for critical dimensions. In this work, we demonstrate broadband extreme-ultraviolet high-harmonic-generation 0th order scatterometry that delivers fast, reliable, non-destructive metrology for structures with at-wavelength features and delivers sensitivities and accuracies to details far below the diffraction limit.”

RR1 - 8 “[…] and the morphology reconstruction protocol hereby presented makes this approach robust against fabrication imperfections.” Maybe remove this sentence. Multimodalities need to be taken into account. Resist residuals and native oxides will influence the reconstruction.

Authors reply: Thank you for your comment. We convene that can be misleading, especially in the abstract. For this reason, we have decided to eliminate the sentence and reformulate that part of the abstract.

Change: The sentence was removed, and the abstract was changed, to see it please check the above comment (RR1 - 7).

RR1 - 9 “[…] enabling reliable patterning of features down to 3 nm on Silicon wafers.” o I guess this refers to the 3 nm node since smallest half-pitch of 5 nm have been achieved by interference lithography at Paul Scherrer Institute (https://doi.org/10.1039/D4NR01332H)

Authors reply: Yes, we apologize for the generalization. We have decided to expand the sentence and include both the current technology node and the smallest feature printed so far by means of nanolithography.

Change: The sentence has been rewritten as: "The current state-of-the-art fabrication techniques rely on nanolithography, enabling reliable patterning of features down to about 10 nm on Silicon wafers, and leading to the current 3 nm technology node."

RR1 - 10 "Scatterometry offers an alternative path to conventional imaging-based metrology techniques enabling precise characterization of nanoscale structures.". Scatterometry with "conventional" VIS and UV is known as OCD and an integral part of wafer metrology. You make it sound like it is not applied yet.

Authors reply: Thank you for the remark. In the text we were referring to already existing scatterometry approaches and we do not believe we are making any suggestion that it is a completely novel technique, also in virtue of the citations present in the paragraph. However, we value the suggestion and we have decided to include and example of OCD in the main text, including also a quick mention to the limits of OCD in the VIS and UV range.

Change: We have decided to introduce a short paragraph about OCD: "An example is offered by optical critical-dimension (OCD) metrology, widely deployed in semiconductor manufacturing in the visible range[24,25]. In practice, OCD inverts angle/polarization/wavelength-dependent diffraction to access sub-diffraction structural parameters, but its performance can be limited by parameter correlations and stack-dependent sensitivities. Furthermore, small-pitch nanostructures illuminated with visible-UV light don't produce actual diffraction patterns, effectively requiring complex modeling.".

RR1 - 11 "Typically overlooked 0th -diffraction order" Referenced papers Lohr [20,34] and Bahrenberg [21] only use 0th diffraction order at EUV wavelengths. Generally known as 2-theta-scatterometry (in 'conventional' wavelength ranges). Again, you make it sound like it is a completely new concept.

Authors reply: We apologize for the confusion that this statement might have brought. We did not mean to phrase the sentence to claim the novelty of 0th order scatterometry in its concept, or to suggest that the 0th diffraction order is nearly never used.
We want to state that the 0th diffraction order is typically overlooked in the context of broadband scatterometry (with broadband light). This is true as most of the experiments reported in literature, illuminate the nanostructure with monochromatic light. In practice, the experimental approach reported by Lohr et al. uses the wavelength as a degree of freedom, that gets scanned by tuning the source with monochromators to scan the range of interest.
Our approach is profoundly different, as we illustrate our target with broadband light and we detect the broadband spectrum as described in the Methods section.
The literature reports of approaches based on broadband HHG illumination, however these experiments aim to capture the entire diffraction pattern on a camera located in proximity to the sample, thus losing the opportunity to exploit the 0th diffraction order for morphology reconstruction. Examples of this approach are reported in Porter et al. and Ku et al..

Change: We have decided to correct the abstract as follows: “We introduce a table-top High-Harmonic-Generation extreme-ultraviolet scatterometry with broadband illumination. Our method exploits the spectrally resolved 0th diffraction order of the extreme-ultraviolet light, which, while lacking spatial-encoded information, carries valuable spectral information and offers high diffraction efficiency.”

For what concerns the similar claim in the introduction, the sentence was rephrased as:
“In the framework of XUV scatterometry performed with broadband illumination, higher diffraction orders are often favored, as they provide spatially encoded information within the diffraction pattern. Here we utilize the 0th order and show that similar structural information can be obtained from its analysis.”

RR1 - 12 „However, the 0th diffraction order carries spectrally encoded information and provides the largest diffraction efficiency compared to higher orders.” Quite general. This does not account for all materials and geometries, especially when phase-shift masks are considered.

Authors reply: Thank you for the comment. We agree on the fact that the 0th order does not necessarily provide the largest diffraction efficiency. We are going to soften the sentence.

Change: The sentence in the Introduction section was changed as follows: “However, the 0th order carries spectrally encoded information and can provide the largest diffraction efficiency compared to higher orders.”

RR1 - 13 “These two gratings feature lines along two perpendicular directions, allowing access to two distinct diffraction conditions for each grating morphology.” o This sentence is hard to read, especially since the use of several grating morphologies is only introduced afterwards.

Authors reply: We apologize for the intricated sentence. We have decided to make it more readable and leaner, omitting the mention to diffraction conditions.

Change: The sentence was rephrased as: ”These two gratings have two orthogonal periodicity directions, as shown in Figure 2 c) and d).”.

RR1 - 14 “The work presented in the following was carried out on a set of 36 different metrology targets with 700nm pitch, including six different groove heights between 80 and 130 nm and three different CDs.” o Could you state here the size of each structured field an if they are all on the same sample?

Authors reply: Thank you for the comment. To improve clarity we have decided to include the nanostructured fields size and how the targets are divided among different samples.

Change: The text was changed as follows: “The work presented in the following was carried out on a set of 36 different metrology targets with 700 nm pitch, each of which is written in a 400 × 400 µm2 field. We have manufactured six samples with six targets each, with three different CDs, two replicas per target. The samples have different groove heights between 80 and 130 nm”.

RR1 - 15 Figure 1: The SEM image is quite blurry, with astigmatism and not optimal contrast settings, maybe sample can be reinspected or other SEM image picked. SEM image could be misleading if it stays like this. Could grating parameters be extracted for at least some of the structures?

Authors reply: Thank you for the comment. We acknowledge that the SEM image was a bit blurry. We have decided to acquire and put in the paper a new cross section image. We have also added a subfigure to show the parameters extraction from this kind of SEM images.

Change: Figure 1 and its description were changed according to the reviewer's suggestion.

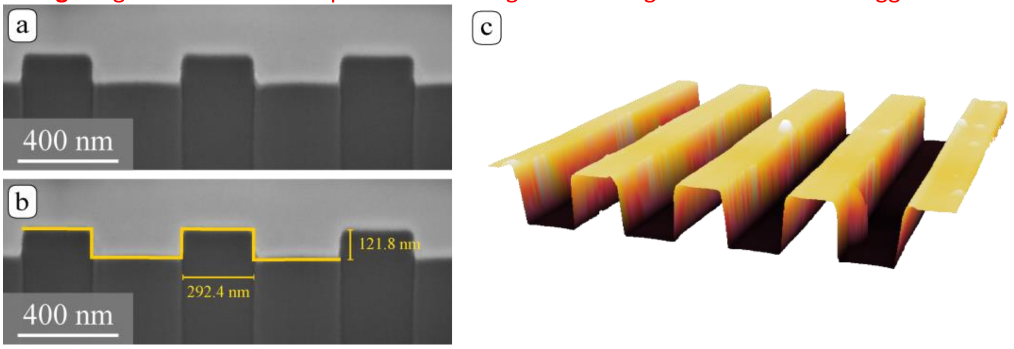

Figure 1. Nanostructured target characterization. a) Grating profile acquired with a SEM imaging. The cross section was obtained by capping a portion of the sample with Ion-Beam-Induced Deposition followed by Ion Milling. b) Cross section fitting with square line profile and parameter extraction. c) AFM measurement (out of plane axis not to scale).

RR1 - 16 "The HHG apparatus can be switched between two configurations to allow some tunability over the XUV spectrum: one dedicated to an ordinary HHG regime, characterized by a discrete harmonics spectrum, and one where the generation conditions are tweaked to obtain a continuum-like spectrum." Maybe show the spectrum in main article for clarity?

Authors reply: Thank you for the suggestion, we have decided to include an additional figure to the main body of the article. We have chosen to add a figure composed of 3 panels. The first two are dedicated to showing the typical spectra used for the experiments for both the discrete and continuous-like generation regimes. The third panel is dedicated to the source stability, also connected to the answer to RR1 – 22.

Change: We have added the following image to the main body of the article, followed by its caption:

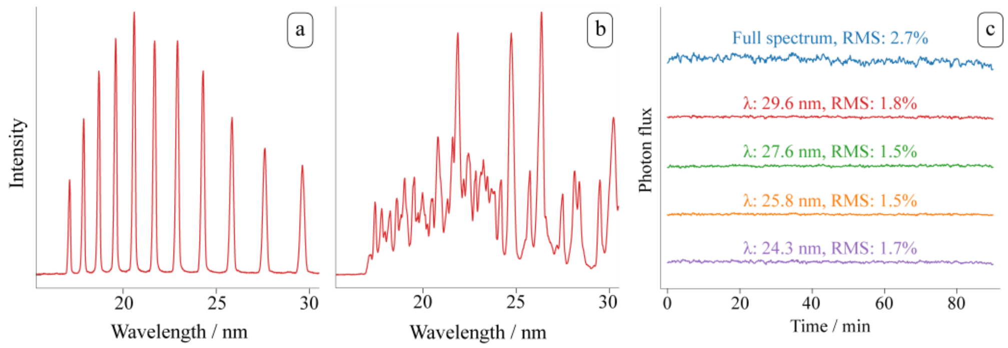

Figure 3. HHG illumination spectrum. a) XUV illumination spectrum obtained in optimal generation conditions, characterized by a discrete spectrum. b) XUV illumination with a continuum-like spectrum. c) Source stability traces for a 90 minutes long measurement in the discrete-spectrum generation regime with a 2s exposure time. We show the stability trace for four different harmonics, and also the stability trace of the total HHG spectrum.

RR1 - 17 "A gold-coated toroidal mirror focuses the XUV beam onto the sample with a spot size of approximately 50µm." Is the beam divergence known, and considered during RCWA simulations?

Authors reply: The beam divergence of the XUV light was not precisely measured but we have characterized its upper and lower boundary. For the experiment described in this paper the beam divergence was estimated to be around 4.7 mrad. In this context, we have the divergence to be negligible, and it was not considered in the RCWA simulations. We have verified the impact of this approximation by simulating the uncertainty on a diffraction spectrum generated by a divergence angle of 4.7 mrad and this turned out to be negligible compared to the uncertainty deriving from the experimental noise.

Change: None.

RR1 - 18 "The HHG beam impinges on the sample with a 78◦ angle of incidence [...]" What is the accuracy on the incidence angle? Is this considered during RCWA simulations or the influence on reconstruction accuracy?

Authors reply: Thank you for this comment. The angle of incidence (AOI) is a critical parameter in this framework, as it strongly influences diffraction efficiency. Even a small deviation, as little as 0.5°, can introduce a consistent error in morphology reconstruction. For this reason, we employ a well-established routine to calibrate the incident angle θ. We use a SmarAct vacuum-compatible piezo rotary stage operating in closed-loop mode, which provides an angular accuracy of 25 µ° (0.44 µrad).

Calibration procedure

The calibration begins by setting the sample stage precisely to 0° AOI. This is achieved using the attenuated IR driver laser:

- The metallic filter is removed, allowing the beam to propagate to the sample.

- The beam is then back-reflected along its original 180 cm path, passing again through the HHG gas cell.
- The 0° AOI is defined as the stage position for which the amount of back-propagating light coupled back into the gas cell is maximized.

This procedure relies on the small acceptance angle of the toroidal mirror and the limited aperture of the gas cell (~80 µm diameter), which together provide a sensitive measure of alignment.

Experimental AOI settings

The 78° AOI used in the experiment is set and measured by the closed-loop controller. Reproducibility tests show a standard deviation of 0.002° (34 µrad). The effect of AOI uncertainty was included in the RCWA simulations. However, in the morphology reconstruction, it was found to be negligible, contributing on average less than 1% to the total uncertainty.

Change: The 'Experimental setup' section of the main article was modified to include "The HHG beam impinges on the sample with a 78◦ ± 0.002◦ angle of incidence".

We have decided to further explain the calibration procedure in the 'Supplementary methods' document, with the addition of the following paragraph: "Calibration of the Angle of Incidence The angle of incidence (AOI) plays a critical role in determining the diffraction efficiency. Even small deviations—as little as 0.5◦, can introduce systematic errors in morphology reconstruction. To minimize this effect, we implemented a dedicated calibration routine for the incident angle θ . A SmarAct vacuum- compatible piezo rotary stage (closed-loop operation, angular accuracy 25 µ◦) was used to control the AOI. Calibration was performed by aligning the sample stage to 0◦ AOI as follows: the attenuated IR driver laser was directed onto the sample after removal of the metallic filter and subsequently back-reflected along its original 180 cm path through the HHG gas cell. The 0◦ AOI position was defined as the stage setting that maximized the amount of light back-coupled into the gas cell. This method exploits the small aperture of the gas cell to provide a sensitive alignment condition. In the experimental configuration, the 78◦ AOI was set and measured using the closed-loop controller. Reproducibility tests yielded a standard deviation of 0.002◦."

RR1 - 19 "The 0th order is subsequently diffracted by a flat-field XUV blazed grating at - grazing incidence onto an XUV sensitive CCD with a sensor cooled to a temperature of −90°C."

Does the spectrometer have an entrance aperture/slit? Or is the mentioned pinhole used as entrance aperture? What is its width?

Quite low temperature of -90°C, could you give a reason for this setting? Exposure times are stated with two seconds only.

Authors reply: Thank you for the question. The XUV spectrometer does not have an entrance slit. We are using a dispersive grating with a focal length of 235 mm (3 deg incident) and we are in a 1:1 imaging configuration. The pin-hole has a diameter of 2 mm. The pin hole is followed by a metallic filter to ensure good stray light suppression.

The camera was operated at -90°C as the setup is generally used for spectroscopy and usually operating at this temperature. As the noise performance of the camera at this temperature are well characterized and offer reliable operation we have chosen not to change this setting.

Change: None.

RR1 - 20 How did you validate during measurements, that you actually hit the azimuthal angles of 0° and 90°? Can you give a measurement uncertainty and is it considered during RCWA simulations or the influence on reconstruction accuracy?

Authors reply: Thank you for your comment. We indeed verify the alignment of our sample, as the azimuthal angle plays a crucial role in the diffraction conditions. We determine and verify our angles using two complementary procedures.

Firstly, during sample mounting in the experimental setup, the sample is placed in a custom holder designed to minimize angular offsets. We verify the alignment using a green laser diode that propagates along both the IR laser and XUV paths. In this context, we exploit the weak diffraction pattern of the grating in planar geometry and align this pattern to a reference mark placed approximately 35 cm from the sample. With this approach, we ensure that the sample stage has no tilt and that the azimuthal angle offset from 0° (planar diffraction) is minimized. Using this method, the uncertainty on the azimuthal angle is about 0.5°.

Secondly, once the sample is loaded and the system is under vacuum, we perform a referencing routine in which we scan the sample with the XUV beam to identify the exact locations of the targets. The targets around each sample follow a grid-based layout established during the E-Beam Lithography process, with nanometer-scale accuracy. This allows us to derive the two-dimensional relative positioning of the targets, from which we obtain a precise estimate of the azimuthal angle. Considering that the most distant targets are separated by 8 mm, and given the accurately characterized beam spot size in the focal plane, we can determine azimuthal angles with a precision of 0.1°.

For the experiments presented in this work, we chose to use simulations at exactly 0° and 90°. To justify this, we considered a maximum tolerance of 0.3° on the actual azimuthal angle. We verified the effect of this angular offset and concluded that it does not have an observable the reconstruction procedure.

We would like to underline that, following your suggestion in comment RR1-3 we have introduced a term to the variance to account for possible sources of systematic errors like this one.

Below we show an example of the impact of a 0.3° offset on the 0th-order spectra:

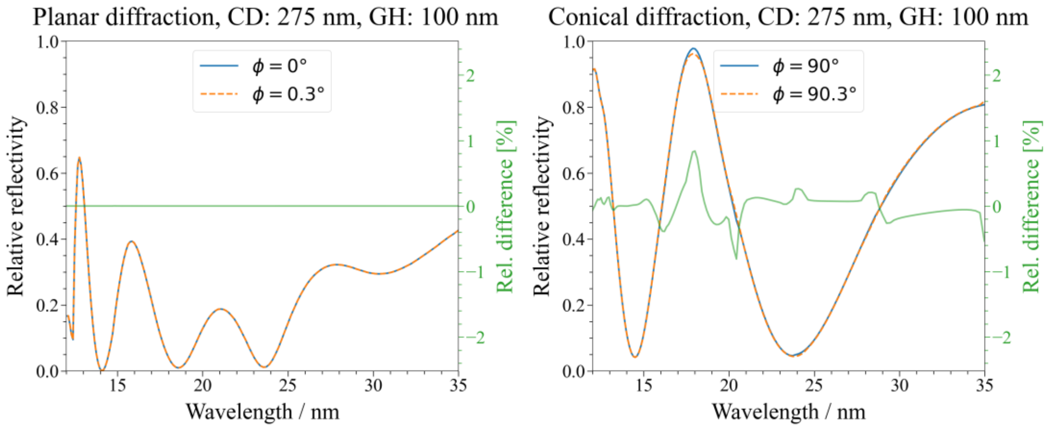

Change: We have decided to expand the text in the main article to the following: "The orientation of the targets was chosen to match the azimuthal angles of ϕ = 0◦ and ϕ = 90◦, where ϕ is defined as the angle between the target periodicity direction and the beam propagation direction projected on the sample plane. The sample orientation was aligned complying with a 0.3◦ tolerance, verified experimentally with a raster scan of the metrology targets. The tolerance was chosen evaluating the impact of misalignment of the azimuthal angle with RCWA simulations.".

RR1 - 21 "By focusing on relative changes, the analysis becomes more straightforward and less prone to systematic errors." Systematic errors like the one on incidence angle, still should be non-negligible, so this statement at least is not correct in all accounts.

Authors reply: The reviewer is right, and we thank them for pointing this out. We of course referred to systematic errors, e.g. the wearing and oxidation of metallic filters due to prolonged exposure to the driving laser. We will adjust the statement.

Change: The text in the main body of the paper was changed to: "By focusing on relative changes, the analysis becomes more straightforward and less prone to some systematic errors".

RR1 - 22 "The experiments hereby presented were carried out with a 2s exposure time and by averaging over five spectra per dataset." Could you please give a statement on the statistics if only five spectra are used? Maybe sufficient if the source is running very stable.

Authors reply: Thank you for the comment. We have decided to introduce in the main body of the paper an image that contains also stability traces of the harmonics during a measurement, Figure 3 – c). As shown in this plot, the harmonics present RMS fluctuations below 2%. For independent fluctuations, the comprehensive uncertainty is reduced by a factor of $\sqrt{n}$. With this consideration in mind, we have chosen a sample with $n = 5$ to push the impact of the spectral fluctuations below the 1% threshold.

Change: Figure 3 – c) was added to display the stability trace of the HHG source calculated form the data of one of the measurements.
Furthermore, a sentence was added to the main text to make the choice of averaging over 5 spectra clearer:
"The experiments hereby presented were carried out with a 2 s exposure time and by averaging over five spectra per dataset. This choice was made to push the impact of spectral fluctuations below the 1% (RMS) threshold."

RR1 - 23 Besides Diffraction of the different grating morphologies, was the reference reflectance of the unstructured sample also simulated to create the rel. reflectivity change and if so, also by RCWA?

Authors reply: Thanks for the question. Yes, the reference reflectance was also computed deploying the RCWA code, starting from the refractive indexed acquired through the CXRO database.

Change: We have added a sentence to specify the origin of the refractive indexes: "The complex refractive indices n + ik for each material were acquired from the CXRO (Center for X-Ray Optics) database[34, 35]".

RR1 - 24 Figure 3 As the simulations were done in larger increments, isn't the data shown here interpolated, and if so: What approach was used to interpolate between datasets? How is this considered during parameter reconstruction.

Authors reply: Thank you for the question. Figure 3 shows the simulated data with a shading option native of the Matplotlib module used for the plotting, i.e. the Gouraud shading. The crude image would look like the following:

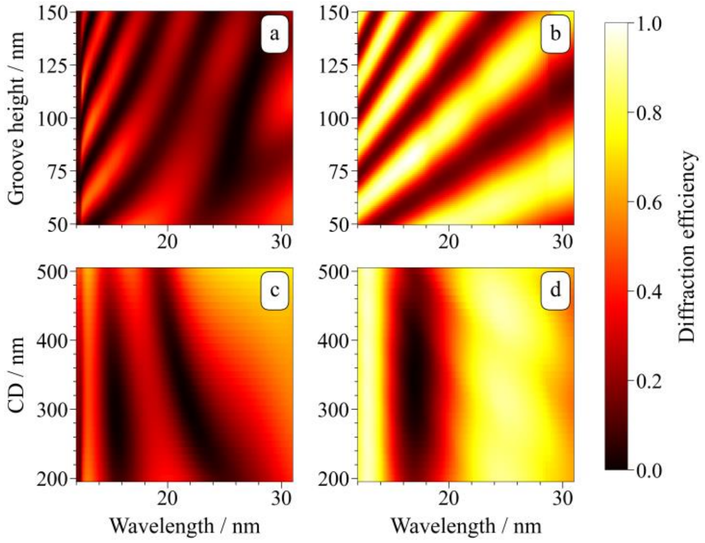

In contrast with the one in the main text of the article:

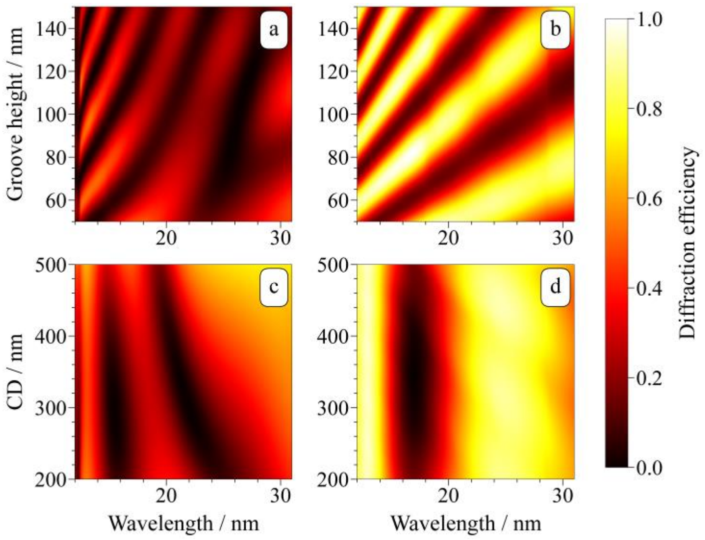

We would like to emphasize that no interpolation was used for the morphology reconstruction.

Change: None.

RR1 - 25 Figure 4 Indicate linewidth and CD in the plots of Figure 4 for clarity.

Authors reply: Thanks for the suggestion. We have labeled the values of critical dimension (CD) and groove height (GH) in the plot.

Change: Figure 4 was modified as requested, here it follows:

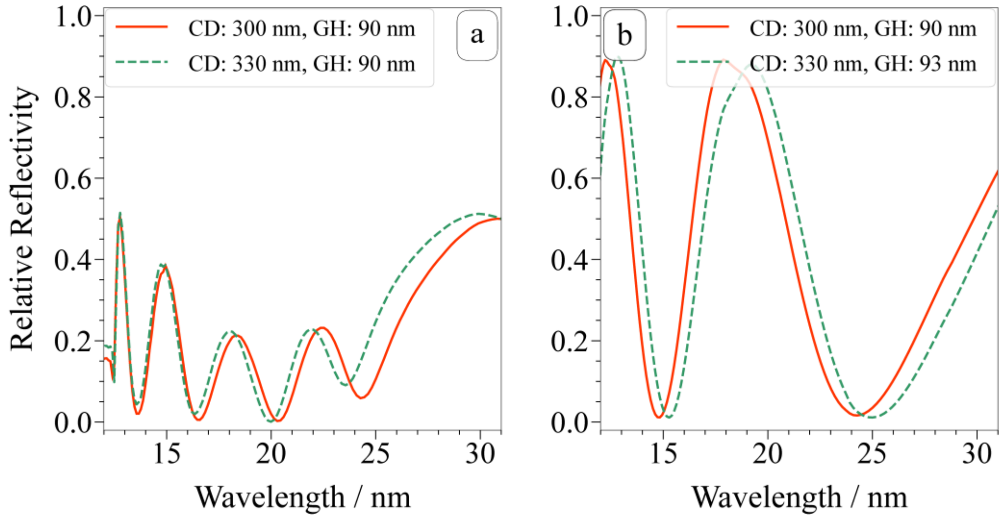

Figure 4. Comparison of the RCWA simulated spectral relative reflectivities for small morphology changes in both diffraction regimes. a) Planar diffraction: comparison of two structures that differ in CD by 30 nm. b) Conical diffraction: comparison of two structures that differ in GH by 3 nm.

RR1 - 26 "Conversely, under conical diffraction conditions the diffraction efficiency profiles are largely independent of CD, as shown in the 3(d) […]" Is that generally the case or only because you use radiation at a much smaller wavelength than the pitch/CD size? --> Less diffraction, less sensitivity. The sensitivity to height comes additionally from "thin film/Bragg interference" where your wavelength matches the probed height difference much better.

Authors reply: We thank the referee for this comment. In our view, the near-independence of the conical diffraction spectra on CD is a general feature for conical diffraction from nanostructures with rectangular cross-sectional profile.

To verify this, we have performed additional RCWA simulations for gratings with a 100 nm pitch and compared them with the simulated data for 700 nm pitch. The results are shown in the two new figures below. In both cases, we plot the relative reflectivity as a function of wavelength for three different CDs (duty cycles). All gratings have a groove height of 70 nm, and the conical diffraction geometry is the same as in the experiment.

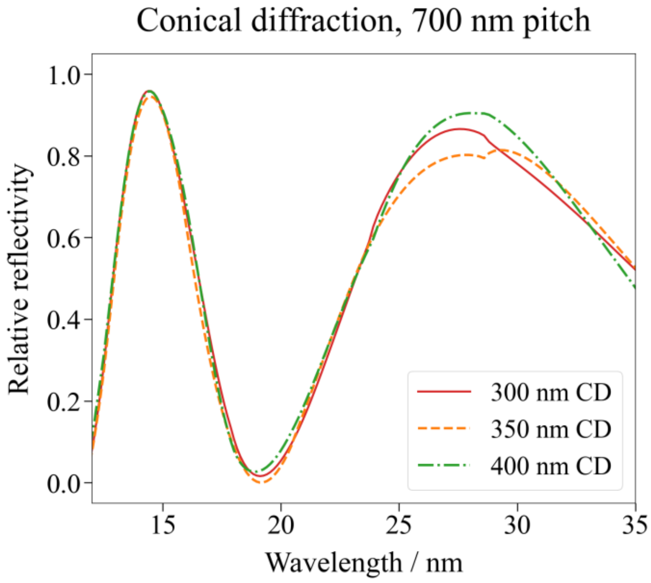

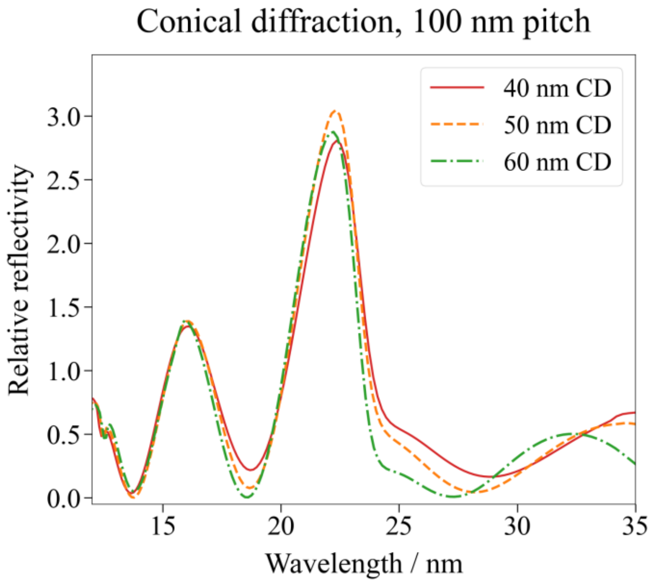

From these plots it can be seen that, for both pitches (and corresponding feature sizes), the influence of CD on the conical diffraction spectra is small compared to the overall spectral modulation. This confirms that, under our conical diffraction conditions, the diffraction efficiency profiles are only weakly dependent on CD.

The strong wavelength dependence of relative reflectivity mainly arises from diffraction, which inherently includes the phase accumulated over the line height and the interference of fields within the grating profile. Variations in CD act only as a relatively small perturbation on this dominant response.

Change: None.

RR1 - 27 “This is an intrinsic limit for 0th order scatterometry that affects the reconstruction precision of transverse features in general, that can only be overcome by collecting more information from other diffraction patterns, such as the second orders for accurate CD reconstruction.” Can you really say that? See comment on independence between diffraction efficiency and CD.

Authors reply: We thank the referee for this comment and for pointing out that our original wording was too general. Our intention was not to claim a fundamental limitation of all possible 0th-order scatterometry schemes, but to describe a limitation that is specific to the approach presented in this work. In our experiment we only use the spectrally resolved 0th order at a single (large) incidence angle, for square cross-section Si gratings.
However, our statement is consistent with results from soft-x-ray scatterometry that use the full diffraction pattern, where multiple higher orders provide strong and well-decorrelated sensitivity to lateral parameters with sub-nanometer accuracy (C. Porter, et al. "Soft x-ray: novel metrology for 3D profilometry and device pitch overlay." *Metrology, Inspection, and Process Control XXXVII*. Vol. 12496. SPIE, 2023). This behavior can be understood from a Fourier-space perspective, where higher diffraction orders probe higher spatial-frequency Fourier components of the structure, which are much more sensitive to lateral dimensions.

Change: Following the referee suggestion we have decided to modify the sentence as follows: “In the context of the experiments hereby presented, reaching at and below single nanometer CD precision would require additional information from other diffraction channels, for example spectrally resolved higher diffraction order.”

RR1 - 28 Do I understand correctly, that you used the LSR only to compare experimental data with data from your prepared database. If so, how did you extract the morphology parameters, if they are set between two dataset points? (Related to the question on the interpolated (?) Plot in Figure 3).

Authors reply: Yes, your understanding is correct. The LSR algorithm was developed to treat ambiguous cases as residuals arrays containing two absolute minima sharing the same value. In such case the routine warns the user with an error message and provides both the outputs connected to those points, but this did not happen for any of the measurements we have performed.
This is surely a problem to be addressed for future works, specifically for more complex target geometries and larger amount of parameters. This is one of the reasons behind our choice of moving to a reconstruction approach based on Simulation Based Inference, please see reply to comment RR1 – 6.

Change: None.

RR1 - 29 “Each peak was individually fitted to a Gaussian curve, and the area under the curve within the 1-sigma width was considered as the signal value for each datapoint.”

a) Doesn’t a peak in the HHG spectrum not have an intrinsic width, that is made of radiation with an according bandwidth? What is the bandwidth of your peaks?
b) Is that then your wavelength accuracy?
c) What would be the possible accuracy of your used spectrometer?
d) Don’t you lose important wavelength resolution by your method?
e) What did you do, when using the HHG in the mode of quasi-continuous spectra?

Authors reply: Thank you for the questions. We took the liberty of organizing the question (and therefore the answer) in subpoints to help readability.

a) Yes, your statement is correct, every harmonic has an intrinsic linewidth. We assess this while calibrating the wavelength axis of our detector. For the setup configuration described in the paper and the spectra obtained during the measurements, the harmonics show a consistent linewidth of 126 meV ± 22 meV, which translates to the following bandwidth for the spectra expressed in wavelength:

```
Harmonic peak: 32.0 nm --> σ: 0.104 nm
Harmonic peak: 29.6 nm --> σ: 0.089 nm
Harmonic peak: 27.6 nm --> σ: 0.077 nm
Harmonic peak: 25.8 nm --> σ: 0.068 nm
Harmonic peak: 24.3 nm --> σ: 0.060 nm
Harmonic peak: 22.9 nm --> σ: 0.053 nm
Harmonic peak: 21.6 nm --> σ: 0.047 nm
Harmonic peak: 20.5 nm --> σ: 0.043 nm
Harmonic peak: 19.5 nm --> σ: 0.039 nm
Harmonic peak: 18.6 nm --> σ: 0.035 nm
Harmonic peak: 17.8 nm --> σ: 0.032 nm
Harmonic peak: 17.0 nm --> σ: 0.029 nm
```

b) This is not our wavelength accuracy, that is determined by the calibration process. We can firmly state it as the same setup was used before to measure line-shapes with widths of few tens of meV (paper). However, we have evaluated the impact of these sigma values on the reconstruction, and we cannot observe any significant effect. This is plausible and the spectral shifts associated to morphological changes are substantially larger that these sigma values.

c) The spectral resolution of our spectrometer is equal to 44.9 ± 0.9 meV, which corresponds to 0.015 nm at a central wavelength of 20.5 nm.

d) For the reasons given above, there is no large loss of resolution by our method. However, it could be that our method does not fully exploit the spectral resolution offered by the spectrometer. On the other hand, the scope of this paper is not to make a detailed characterization of our experimental setup, but it is to establish the 0th order scatterometry based on HHG sources as a reliable and robust metrology tool.

e) The usage of spectrally quasi-continuous illumination was possible by altering the generation conditions of the HHG source. The relative reflectivity data was extracted with a element-by-element division along the spectral axis. In this context every element (pixel) of the collected spectra corresponds to a bandwidth of about 20 meV. To further clarify this point, a short paragraph has been added to the supplementary section.

Change:

"In the first case, the traditional phase matching conditions for HHG are met, while in the later case the ionization rate in the gas is increased by higher field intensities. This leads to modulation of the field which reduces the overall harmonic yield in favor of creating a much more continuous spectrum. Switching between the two HHG processes is achieved by controlling the initial diameter of the beam using a pinhole before the focusing lens, while the focusing position remains the practically the same."

RR1 - 30 Did you only consider statistical uncertainties in your data? What about all possible systematic errors? (Angle accuracy, long-term source stability, straylight, etc. What about it's influence on reconstruction accuracy?

Authors reply: Thank you for raising this important point. In addition to statistical uncertainties, we have carefully considered the relevant sources of systematic error in our measurements. Angle accuracy is addressed in detail in our responses to RR1–17, RR1–18 and RR1–20, and the long-term stability of the source is discussed in RR1–22.

With regard to stray light, we employ a dedicated metallic filter placed directly in front of the spectrometer to suppress unwanted radiation. We further analyze a representative subset of the recorded camera images to verify that no signal above the signal-to-noise ratio threshold (SNR = 1) is present outside the harmonic peaks. The total acquisition time for a typical measurement is on the order of a few minutes, which is short enough that slow drifts in laboratory conditions (e.g. temperature and humidity) do not play a significant role.

Other potential systematic contributions, such as occasional mechanical vibrations of the optical table, are expected to have a negligible impact on our results. We also emphasize that the main objective of this work is to demonstrate 0th-order scatterometry with HHG sources as a robust metrology approach that does not rely on absolute calibration or perfectly ideal measurement conditions.

In principle, systematic errors that exclusively affect the 0th-order diffraction efficiency could degrade reconstruction accuracy. However, such effects can be straightforwardly identified by measuring a well-characterized reference sample. In fact, the residual layer discussed in the manuscript is a clear example of such a systematic effect. At the start of our experiments, we were not aware of this layer, but the measured data showed unexpected behavior that prompted a more detailed investigation of the sample. In this sense, while systematic errors can influence reconstruction accuracy, the resulting deviations in the measured response also provide a valuable diagnostic that can reveal their underlying cause.

Change: None.

RR1 - 31 Figure 5 In the figure caption the order between CD and groove height is switched for the plot in c), this could be easily adjusted.

Authors reply: Thanks for highlighting the mistake in the text order, we apologize for it.

Change: The caption of Figure 5 was corrected to "c) Sample with 400 nm CD and 92 nm groove height".

RR1 - 32 Figure 6 Could you comment on the visible multimodalities. Why is AFM used here? I guess the cross-sectional SEM could give more realistic insight. Either way, how many lines have been considered to estimate the reference values?

Authors reply: We have used Atomic force microscopy instead of SEM cross-sections for groove height characterization for 2 main reasons:

1. Performing and acquiring an SEM cross section is a slow process. Every cross section requires ion-assisted Tungsten (W) deposition over a 7 um x 4 um area to ensure a good

exposure over at least 5 grating lines (with a bit of overhead). This has then to be milled by means of focused ion beam, and finally imaged. This procedure takes nearly one hour and should be repeated multiple times around each grating to ensure groove height uniformity. Considering we had characterized 36 targets, composed of 72 gratings, this approach would be highly time consuming (at least 144 hours) and expensive. On the other hand, AFM is significantly faster, as the scan of a portion of a grating takes 4-7 minutes. Atomic force microscopes can also offer very high out-of-plane resolution, down to the picometer.

2. SEM cross-sectional images require to permanently damage the samples, as metal deposition and milling are in order to perform the characterization. On the other hand, AFM is substantially less invasive, specially in the so called 'Tapping mode', causing minimal damage to the sample surface.

10 different sample scanning over 5 grating pitches were used for the groove height characterization. The groove heights were evaluated independently for every grating line, and then the results averaged.

Change: None.

RR1 - 33 "This approach, however, cannot deliver accuracy in feature reconstruction better than the simulation mesh spacing, which was 1 nm for the groove height and 10 nm for the CD in the library used in this work."

1. previously you wrote about spacings of 2nm / 25nm respectively. Which one is correct? If your accuracy cannot be better than this, why do you give smaller values in your plots in Fig. 6 & 7?
2. Does the Std in the plots only come from taking the mean of all samples? I think that you have to expect variations between different samples (due to small fabrication variations) so taking this value as a measure for uncertainty is not reliable.
3. Your first estimation on accuracy should be derived by the width of your euclidian distance distribution and only when that would be smaller than your data set steps, you can use these values as minimal accuracy.

Authors reply:

1. We apologize for the mistake, the increments are 1 nm and 10 nm respectively. The wrong numbers came from one of the first versions of this paper.
2. Thank you for the comment which is correct. We have been careful with it. It might be easier to understand how the sample calibration values were averaged with the correction we have completed following the reviewer comment RR1 – 14 that explains in detail how the samples are distributed among different substrates and different morphological parameters. In detail, all the targets that share the same groove height were fabricated on the same Silicon substrate. This implies that the etching took place at the same time for all the targets, and nearly in the same position, thus guaranteeing very similar groove heights as manifested by the standard deviation of the characterization reported in Figure 6. A similar argument applies to the CD reconstruction in figure 7. The reason why we have opted for averaging of the independent parameters characterization is that the wiggle room of the reference value was smaller than the actual library granularity.
3. Thank you for your suggestions, and your remark is essentially correct. For this reason we have moved the data analysis to a full chi-squared LSR framework. The matter of accuracy is discussed in detail the in the answer to the comment RR1-3 above.

Change:

1. The text was changed to: “The library was designed to cover a parameter space that includes gratings with groove heights ranging from 60 to 150 nm, in 1 nm increments, and critical dimensions (CDs) between 200 and 500 nm, in 10 nm increments.”
2. None.
3. We added to the discussion the following paragraph: “It is important to note that the morphology reconstruction is performed by comparing the measured reflectivity spectra against a discrete RCWA library, defined on a finite parameter grid. Therefore, the library sampling determines the numerical resolution of the brute-force search and sets the minimum spacing between candidate geometries. However, this does not constitute a limit as the reconstruction uncertainty is not directly connected to the library mesh size. Instead, it is extracted from the $\chi 2$ loss landscape through confidence intervals derived from the condition $\Delta\chi 2 = 1$ (for a single parameter), which reflects the curvature of the loss function and the effective noise level of the measurement. This implies that the confidence intervals provide an intrinsic uncertainty estimate of the least-square inference that can be smaller or larger than the grid spacing, which mainly depends on the local sensitivity of the $\delta R\lambda$ (p) signal.”.

RR1 - 34 “The extra cleaning procedure proved effective in reducing the persistent resist layer, as characterized by means of XPS.”

1. Was the contamination not detected already when you used XPS to identify the SiO-top layer of the samples? If not, why?
2. Are all previous measurements used for morphology reconstruction done with contaminated samples (in the supplements the cleaning step is named)? If no, why bring this topic up so late and not beforehand? If so, why couldn’t the measurements und reconstruction be redone with cleaned samples (as it was done with the sample in fig.8 c)? (beamtime is an argument I guess)

Authors reply: Thank you for your comment and for the opportunity to better explain this topic. The reason why we have decided to include the story of this resist residue layer is not only to accurately describe our sample and explain the offset of the experimental data with respect to the simulations, but also to showcase the extraordinary sensitivity of 0-th order scatterometry to systematic surface contaminations.

1. The first XPS inspection was performed only after some initial scatterometry measurements that revealed a large systematic offset with respect to simulations. At that stage, XPS was used exclusively to quantify the native SiO_2 thickness by analyzing the Si-2p region. The C-1s region was not examined in detail. At that point, we had not yet identified the presence of a structured, trench-localized resist residue.
   This residue was first noticed in a later tilted top-view SEM inspection (similar to the SEM image included in the paper, Figure 9a), where it appeared as a thin and spatially uniform opaque film on top of the substrate. Only after recognizing this feature in SEM and linking it to the reflectivity offset did we decided to perform the extra cleaning step, whose result is shown in figure 9b in the paper. Additional XPS scans were repeated on the cleaned samples, with an explicit analysis of the C-1s region. Those later measurements showed the presence of persistent carbonaceous residue, composed of sp^2/sp^3 hybridized species. So, the reason the contamination was not reported earlier is not a limitation of XPS sensitivity, but simply because we did not initially perform any analysis around the C edge as we were not expecting resist residue at this stage of the process.

2. All results reported in the main manuscript (scatterometry measurements, reconstructions, and reference metrology) were obtained after an additional RCA + acid piranha cleaning step. As a result, no morphology reconstructions reported in the paper rely on unwashed samples. The only data shown from before the additional cleaning are the comparative spectra in Figure 9c, whose purpose is solely to document the effect of the residue on the reflectivity. All reconstructions, as well as the AFM/SEM characterization used as benchmarks, correspond to samples that underwent the extra cleaning procedure.

Change: We have followed the suggestions from the reviewer, and we have introduced the presence of the residue layer in the Methods section, expanding an existing paragraph as follows: "After the fabrication process, the samples were characterized via X-Ray Photoelectron Spectroscopy (XPS) which revealed a 1.2 ± 0.1 nm thick native oxide layer on the surface. The thickness was measured by comparing the atomic percent ratios of the native oxide and pure Si signals from the Si-2p core region32. The XPS characterization also detected the presence of some organic contamination in the shape of a fractional monolayer, a leftover from the resist used in the nanofabrication process (more details in the Supplementary Methods)".

Also the Discussion session was expanded to better explain the storyline of this resist residue layer: "From the results presented in Figure 6, a systematic mismatch between measured and simulated relative reflectivity can be observed. We traced this discrepancy to a thin carbonaceous residue left after resist stripping, distributed uniformly over the nanostructured regions. This residue was first identified through tilted SEM imaging (Fig. 9-a), while initial XPS measurements was only focused on Si-2p and O-1s for oxide characterization. Once the residue was recognized in SEM, an additional RCA + acid piranha cleaning step was carried out, followed by XPS measurements targeting the C-1s region. All scatterometry results and morphology reconstructions reported in this manuscript were performed after this extra cleaning step. Pre-cleaning measurements are shown only in Fig. 9 as a comparison illustrating the influence of the residue on the spectral response."

Ultimately, also the Supplementary Methods section concerning the Nanostructured samples have been expanded to include a note about the residue layer: "After the fabrication process, the samples were characterized via X-ray Photoelectron Spectroscopy which measured a 1.2 ± 0.1 nm thick native oxide layer on the surface. The evaluation was conducted by comparing the atomic concentrations of the silicon oxide and silicon peaks in the Si-2p region within the binding energy range of 96 − 110 eV. We note that the initial XPS measurements were performed at a stage when only the Si-2p and O-1s regions were examined for quantification of the native oxide. At that time, the presence of resist residue was not yet suspected, and the C-1s region was not analyzed in detail. The residue was identified later through tilted SEM imaging, which revealed a thin, spatially uniform film on the structured regions. This observation prompted the extra cleaning procedure and a subsequent XPS analysis explicitly targeting the C-1s region on the cleaned samples, confirming the presence of persistent carbonaceous material in the form of sp2 and sp3 hybridized species."

RR1 - 35 Lohr et al. is referenced twice (date is wrong one time).

Authors reply: We apologize for the mistake. We will correct it.

Change: The double reference with the wrong data was eliminated.

RR1 - 36 Number of references (52) does not match references in text (Number only goes up to 45) - Reference 48 is just 'Optica Publishing group'

Authors reply: We apologize for the mistake. After careful check it turned out that there were indeed 2 unused references, an empty reference and one incorrectly referenced in the text.

Change: We have corrected the reference section by eliminating the empty items and deleting the unused ones.

RR1 - 37 "After the fabrication process, the samples were characterized via X-ray Photoelectron Spectroscopy which measured a 1.2±0.1nm thick native oxide layer on the surface." The resist residues are not mentioned here, a bit confusing. How does this lineup in the storyline?

Authors reply: Thank you for the remark. We agree on the need for a more detailed explanation of the surface characterization results. We will also include the result from the characterization of the residue layer. The details in the storyline are explained in the above **RR1 – 34** point.

Change: The above-mentioned sentence was followed up with another sentence as follows: **"**After the fabrication process, the samples were characterized via X-Ray Photoelectron Spectroscopy (XPS) which revealed a 1.2 ± 0.1 nm thick native oxide layer on the surface. The thickness was measured by comparing the atomic percent ratios of the native oxide and pure Si signals from the Si-2p core region32. The XPS characterization also detected the presence of some organic contamination in the shape of a fractional monolayer, a leftover from the resist used in the nanofabrication process.

RR1 - 38 Maybe add other/better SEM images.

Authors reply: We have already taken action in the comment RR1 – 15.

Change: None.

RR1 - 39 Maybe combine with some AFM scans

Authors reply: Thank you for the suggestion. An AFM scan would not add technical detail to the content of the paper, however it can be valuable in helping the reader understand and visualize the nanostructure morphology in 3 dimensions.

Change: The 3D plot of an AFM scan was added to Figure 1, specifically in subfigure c). here follows the image.

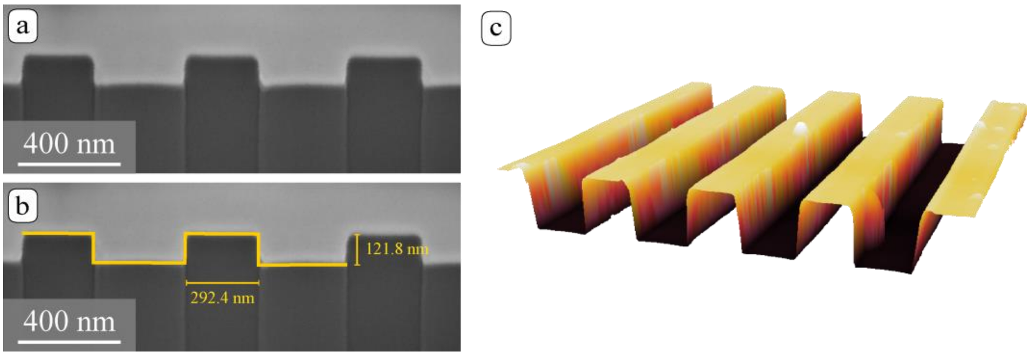

Figure 1. Nanostructured target characterization. a) Grating profile acquired with SEM imaging. The cross section was obtained by capping a portion of the sample with Ion-Beam-Induced Deposition followed by Ion Milling. b) Cross section fitting with square line profile and parameter extraction. c) AFM measurement (out of plane axis not to scale).

Report of Referee #3:

RR3 - 1 The author presents their scatterometry work using a broadband extreme ultraviolet source for establishing metrology on every shrinking critical dimension in nanofabrication. The article is divided into two main sections. A rather small section that describes the light source and a much longer section that describes how the scatterometry samples and analysis. The samples have rather dimensions relative to the aim of providing a metrology solution on critical dimension for state of the are nanofabrication. It is possible to characterize the presented samples without EUV light.
The presented scatterometry analysis method is well known and has been reported in many articles before. The main significance of the article is the light source. Working in the EUV regime using a tabletop source higher harmonic generation from a gas cell is a significant step forward from synchrotron source and laser-produced plasma source at 13.5-nanometer. I therefore suggest that the focus of the article should be shifted more towards the light source.

Authors reply: We would like to thank the referee for this valuable suggestion. We have revised the Methods section that describes the experimental apparatus, and shifted the focus towards the High-Harmonic generation source. We have extended the description of the setup, adding also relevant information about the HHG flux and the focused beam size characterization process. We have introduced a new Figure to showcases the broadband spectra achievable with our HHG source (both in discrete and quasi-continuous coverage) and the source stability on the time-frame typical of these 0th order scatterometry measurements. Furthermore, we have included a paragraph about how we switch between discrete and continuous harmonics is added in the supplementary section.

Change:

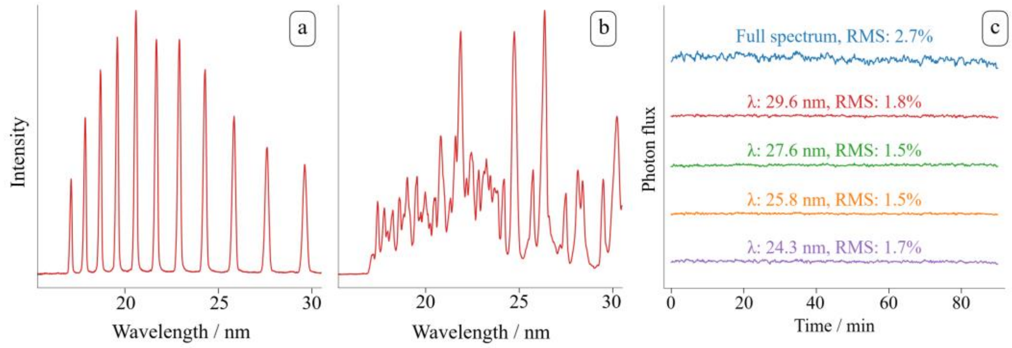

Figure 3. HHG illumination spectrum. a) XUV illumination spectrum obtained in optimal generation conditions, characterized by a discrete spectrum. b) XUV illumination with a continuum-like spectrum. c) Source stability traces for a 90 minutes long measurement in the discrete-spectrum generation regime with a 2 s exposure time. We show the stability trace for four different harmonics, and also the stability trace of the total HHG spectrum.

" A gold-coated toroidal mirror focuses the XUV beam onto the sample. A beam waist characterization was performed in the reflection geometry by using two complementary methods. The first one uses one of the grating targets as the effective edge,34 and the other

one by using a razor blade that was attached at 7◦ with respect to the sample surface. In the first case, scanning the structured surface provides with the intensity distribution (albeit the minimum is no longer at zero) which is then utilized to calculate the beam waist in both the short and long axis of the ellipsoid projection of the beam.

The second method, although closer to traditional beam characterization techniques, poses a notable issue and was performed only to complement the first. The razor can never bem perfectly tangent on the surface of the sample, therefore the beam waist that is probed is effectively different from the real projection on the sample.

The short axis (vertical) was found to be 48 ± 3µm when using the grating as an edge and 50 ± 3µm when using a razor. The long axis (horizontal), was measured at 260 ± 7µm beam waist (using only the first method due to limitations of the razor mounting mechanism) which is reasonably close to the estimated projection of the beam on the sample at 12◦ from grazing. An estimation of the EUV flux on to the sample is performed by taking into account the recorded spectrum and beam propagation factors. The expression used is:

$$\frac{\Phi_{EUV}}{pulse} = \frac{E_{meas}}{f_{propagation} \cdot f_{detection}} = \frac{\frac{E_{total}}{\#_{pulses}}}{T_{Al} \cdot R_{sample} \cdot R_{Col} \cdot \eta_g \cdot \eta_{e-h} \cdot \Phi_{CCD}} \quad (2)$$

where Etotal is the total integrated energy reaching the sensor, #pulses is the amount of pulses calculated by the multiplying the repetition rate of the laser with the total acquisition time of a spectrum, T_{Al} is the transmitivity of the second 200 nm Al filter[35],Ri are the reflectivities of the sample and collection mirror[36], η_g is the first order diffraction efficiency of the analyzing grating[37], η_{e-h} is the electron-hole pair creation efficiency of CCD sensors[38] and Φ_{CDD} is the specific quantum efficiency provided by the manufacturer.

The HHG generated in Ne span well over the Al edge, therefore the estimation should be considered conservative. Nevertheless, a typical spectrum gives us an estimation of approximately 10 pJ/pulse which is consistent with previous calculations performed on
the setup[39]. Considering the above, the fluence on the samples is calculated under the assumption of an ellipsoidal beam at 100 nJ/µm^2 . "

RR3 - 2 The article is in general well written and clearly explained.

The authors claims that the 0'th order is seldom used (forgotten) in scatterometry. This is not correct it is the most used order in spectroscopic scatterometry. This statement should be corrected.

Authors reply: We apologize for the confusion that this statement might have brought. We did not mean to phrase the sentence to claim the novelty of 0th order scatterometry in its concept, or to suggest that the 0th diffraction order is nearly never used. To avoid this confusion we will omit this explicit claim from the abstract.
We want to state that the 0th diffraction order is typically overlooked in the context of broadband scatterometry (with broadband light) in the Extreme-Ultraviolet range. This is true as most of the experiments reported in literature, illuminate the nanostructure with monochromatic light. In practice, the experimental approach reported by Lohr et al. uses the wavelength as a degree of freedom, that gets scanned by tuning the source with monochromators to scan the range of interest.

Our approach is profoundly different, as we illustrate our target with broadband light and we detect the broadband spectrum as described in the Methods section.
The literature reports of approaches based on broadband HHG illumination, however these experiments aim to capture the entire diffraction pattern on a camera located in proximity to the sample, thus losing the opportunity to exploit the 0th diffraction order for morphology reconstruction. Examples of this approach are reported in Porter et al. and Ku et al..

Change: We have decided to correct the abstract as follows: "We introduce a table-top High-Harmonic-Generation extreme-ultraviolet scatterometry with broadband illumination. Our method exploits the spectrally resolved 0th diffraction order of the extreme-ultraviolet light, which, while lacking spatial-encoded information, carries valuable spectral information and offers high diffraction efficiency.".

For what concerns the similar claim in the introduction, the sentence was rephrased as:
"In the framework of XUV scatterometry performed with broadband illumination, higher diffraction orders are often favored, as they provide spatially encoded information within the diffraction pattern. Here we utilize the 0th order and show that similar structural information can be obtained from its analysis."

RR3 - 3 I cannot recommend the article for nature communications in its present form. However, I would encourage the author to resubmit a new manuscript with more focus on the source and with investigation on samples with smaller dimensions.

Authors reply: We thank the referee for the constructive feedback. In the revised manuscript, we have addressed both points raised in this comment.

First, we have expanded the discussion of the high-harmonic-generation XUV source and its role in enabling broadband zeroth-order scatterometry, clarifying how source stability, spectral coverage, and photon flux underpin the sensitivity and robustness of the approach. More detail can be found in the reply to comment RR3-1.

Second, we have extended the experimental study to include nanostructured gratings with substantially smaller pitches of 200 nm and 100 nm. Measurements and quantitative morphology reconstruction for these additional targets are now presented in the main section of the articles. These results demonstrate that the proposed method remains applicable at reduced feature sizes, yielding nanometer-level agreement for groove height and few-nanometer to ~10 nm agreement for critical dimension, consistent with the performance observed for the original 700 nm-pitch dataset.
The measurements of the smallest pitches we are showing are now limited by the sample fabrication tools (e-beam) that are at our disposal in our clean room facility.
We believe that these additions strengthen the manuscript by broadening the demonstrated applicability of the technique while preserving its original focus on broadband zeroth-order XUV scatterometry as a robust, table-top nanometrology approach.

3. **Change**: The text in the main article was expanded with the following additions. Methods: "In addition to the 700 nm pitch targets, a set of 4 nanostructures with pitches of 200 nm and 100 nm was fabricated to probe the applicability of the technique to more aggressively scaled geometries. For each pitch, two independent targets were produced on the same Silicon substrate. We followed the same design and fabrication methodology as for the 700nm pitch structures, with CDs corresponding to 50% duty-cycle."

Results: "Furthermore, we performed additional measurements on gratings with pitches of 200nm and 100nm to evaluate the reconstruction capabilities of this approach at reduced feature sizes. Fig. 7 shows the relative reflectivity spectra measured on representative targets for each pitch, acquired in both planar and conical diffraction geometries and compared to RCWA simulations. These measurements were collected with an incidence angle of 70◦ , as the sensitivity to transverse features is relatively low at 78◦ for these feature sizes. Despite the substantially smaller pitch, the measured spectra exhibit clear and reproducible wavelength-dependent modulations across the probed spectral range. Detailed numbers can be found in the Supplementary Information.

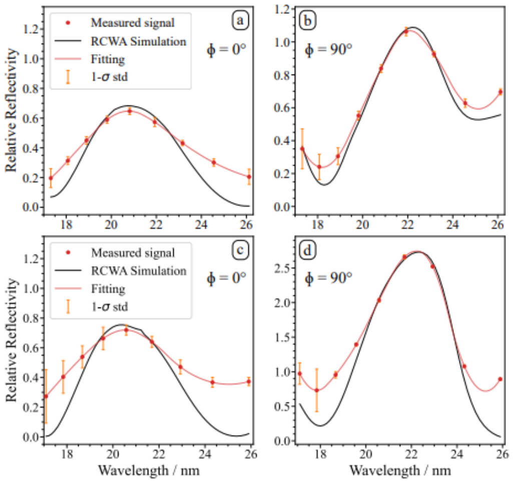

Figure 7. Relative reflectivity measurements for 100 and 200 nm pitch targets. Relative reflectivity measurements for 2 different metrology targets, plotted together with the simulated curves for the two different diffraction regimes. a) and b) report the relative reflectivity measured, respectively, in planar and conical diffraction, for a metrology target with 200 nm pitch and 100 nm nm CD. c) and d) report the relative reflectivity measured, respectively, in planar and conical diffraction, for a metrology target with 100 nm pitch and 50 nm CD. Both targets have a GH of 68 nm."

Discussion: "Morphology reconstruction was also performed for the four small-pitch targets using the same library-based least-squares regression. For the 200 nm pitch targets, the reconstructed groove heights agree with the reference AFM values within 1.1 nm, with combined uncertainties below 1.3 nm. The corresponding critical dimensions are reconstructed within 5−10 nm of the SEM reference values, consistent with the performance observed for the 700 nm pitch dataset. For the 100 nm pitch targets, groove height reconstruction remains accurate at the nanometer level, with deviations from the reference values around 2 nm. The reconstruction of the critical dimension yields 3−7 nm (absolute) bias values, with uncertainty intervals that remain statistically consistent with the observed deviations."

Report of Referee #4:

This paper explores a particular modality of EUV reflectometry/scatterometry for use in measuring critical dimensions and groove depths of gratings in a semiconductor setting. The approach is relatively simple and relies on matching carefully considered measurements to a pre-simulated library of diffraction efficiencies computed using RCWA. The results are surprisingly accurate considering that EUV scattering is usually sensitive to any/all aspects of a sample (intended or not); in part, this indicates a very high quality of sample fabrication. In addition, the paper is well-written with clear figures and proper technical information. I recommend it for publication.

RR4 - 1 In Section 2.2, the authors mention the angle of incidence as 78deg. Much of their audience will likely come from the fields of EUV and soft x-rays, so they could consider specifying that this is 78deg from normal incidence.

Authors reply: We thank the reviewer for bringing this to our attention. The text has been modified accordingly.

Change: The text of the article was changed to: "The HHG beam is focused by a gold-coated toroidal mirror and impinges on the sample with a 78◦ ± 0.002◦ angle of incidence with respect to the surface normal plane."

RR4 - 2 In Figure 3 the authors could consider adding the labels "Planar Diffraction" and "Conical Diffraction" above plots (a) and (b), respectively.

Authors reply: Thank you for the suggestion. Inserting the full "Planar diffraction" labels would be quite time consuming, but we will surely add the labels of the azimuthal angles.

Change: The Figure was changed as suggested by adding to each plot the labels of the azimuthal angles.

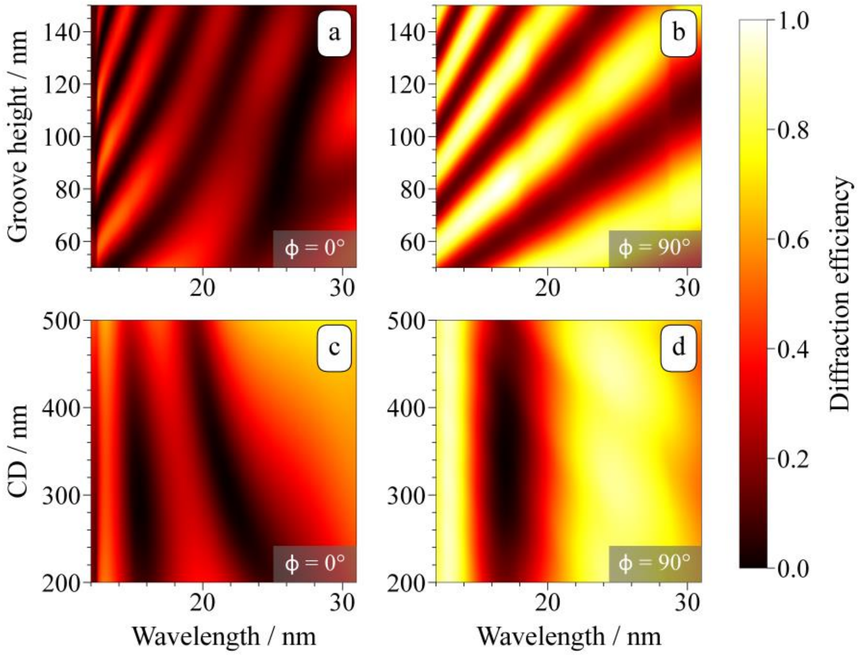

RR4 - 3 supp figure 2 caption is missing a closing parenthesis

Authors reply: Thank you for pointing it out, the correction has been made.

Change: The caption was corrected as suggested.

We again thank the referees for their thorough work and look forward to a final assessment.

Joint report document on Manuscript#: NCOMMS-25-58161

General comments on manuscript

The authors present a sound and well-written publication in the field of nanometrology, often referred to critical dimension metrology. The overall descriptions are detailed in a balanced way, the supplementary information covers even more details. To further improve the quality of the publication we suggest the following aspects that are stated here as well as in the pdf-file. Unfortunately, there were no line numbers, so we referenced them directly in each section.

- Good introduction to a new setup capable of EUV scatterometry with well described setup, process and results. Good use of illustrations and understandable plots.
- Generally, the presented metrology method is not new, and with the presented setup and reconstruction algorithm it is less powerful than already existing setups, still the application of a “no-calibration” method is interesting. But it should be made clearer from the beginning, that a reference measurement (of a non-structured) sample is required, which kind of negates the “no-calibration” approach.
- The uncertainties derived for morphology reconstruction are, in our view, incorrect, and should be revised. They are given as the database increment step, which is mainly based on the fact that the averaging of measurements between different samples (which should be similar but have intrinsic variations e.g. due to fabrication) yields smaller uncertainties, instead of basing the uncertainty on the intrinsic uncertainty of the LSR. Additionally, systematic errors during the experiment (e.g. on incidence angle, azimuthal angle, etc.) seem to be neglected, which in our experience can have a major influence on reconstruction accuracy (small change in incidence angle e.g. can lead to systematic shift of diffraction minima & maxima), even within the “no-calibration” approach.
- Generally, the used sample parameters seem to be no good match for the used wavelength range which could be an explanation why data shows low sensitivity to CD. In my opinion, the diffraction of 10-30 nm radiation at 700 nm is very low. On the other hand, the visible sensitivity to the line height might mainly come form “thin-film” interference between beams reflected of the upper and lower surfaces of the grating. This issue should be addressed in the paper. An additional study on gratings with smaller pitches and the expected sensitivity to CD and line height could show if sensitivity might improve for other grating parameters.
- The amount of measured datapoints used for fitting with the database is a bit low. To be honest, seeing figure 5, fitting a curve to the 11 datapoints that is shifted by one or two nm (in wavelength) wouldn’t be so bad either, which would have a major influence on the reconstructed morphology parameters.
- Due to the “no-calibration” method the current data fitting mainly considers the position of interference maxima and minima and is incapable of fitting the amplitude, which already makes CD reconstruction harder than line height reconstruction. To enlarge the amount of morphology parameters in future research, an extensive study should be done, to determine if the presented method has any sensitivity to additional morphology parameters, and where it will fall short. This should also be made clearer in the Conclusion.

Comments (see also PDF document)

Abstract

- "Most industrial metrology is carried out on periodic structures to increase diffraction efficiencies and lower costs."
    - Sentence a bit hard to read. Maybe it combines several aspects: Most industrial metrology uses periodic structure fields for quality and process control. The diffraction efficiency can be used to determine the test structure geometry that is indirectly linked to the fabrication process of the device structures.
- "[...] and the morphology reconstruction protocol hereby presented makes this approach robust against fabrication imperfections."
    - Maybe remove this sentence. Multimodalities need to be taken into account. Resist residuals and native oxides will influence the recontruction.

Introduction

- "[...] enabling reliable patterning of features down to 3 nm on Silicon wafers."
    - I guess this refers to the 3 nm node since smallest half-pitch of 5 nm have been achieved by interference lithography at Paul Scherrer Institute (https://doi.org/10.1039/D4NR01332H)
- "Scatterometry offers an alternative path to conventional imaging-based metrology techniques enabling precise characterization of nanoscale structures."
    - Scatterometry with "conventional" VIS and UV is known as OCD and an integral part of wafer metrology. You make it sound like it is not applied yet.
- "Typically overlooked 0th-diffraction order"
    - Referenced papers Lohr [20,34] and Bahrenberg [21] only use 0th diffraction order at EUV wavelengths. Generally known as 2-theta-scatterometry (in 'conventional' wavelength ranges). Again, you make it sound like, it is a completely new concept.
- „However, the 0th diffraction order carries spectrally encoded information and provides the largest diffraction efficiency compared to higher orders."
    - Quite general. This does not account for all materials and geometries, especially when phase-shift masks are considered.

Methods

- "These two gratings feature lines along two perpendicular directions, allowing access to two distinct diffraction conditions for each grating morphology."
    - This sentence is hard to read, especially since the use of several grating morphologies is only introduced afterwards.
- "The work presented in the following was carried out on a set of 36 different metrology targets with 700nm pitch, including six different groove heights between 80 and 130 nm and three different CDs."
    - Could you state here the size of each structured field an if they are all on the same sample?
- Figure 1: The SEM image is quite blurry, with astigmatism and not optimal contrast settings, maybe sample can be reinspected or other SEM image picked. SEM image could be misleading if it stays like this. Could grating parameters be extracted for at least some of the structures?

- “The HHG apparatus can be switched between two configurations to allow some tunability over the XUV spectrum: one dedicated to an ordinary HHG regime, characterized by a discrete harmonics spectrum, and one where the generation conditions are tweaked to obtain a continuum-like spectrum.”
    - Maybe show the spectrum in main article for clarity?
- “A gold-coated toroidal mirror focuses the XUV beam onto the sample with a spot size of approximately 50µm.”
    - Is the beam divergence known, and considered during RCWA simulations?
- “The HHG beam impinges on the sample with a 78◦ angle of incidence […]”
    - What is the accuracy on the incidence angle? Is this considered during RCWA simulations or the influence on reconstruction accuracy?
- “The 0th order is subsequently diffracted by a flat-field XUV blazed grating at
- grazing incidence onto an XUV sensitive CCD with a sensor cooled to a temperature of −90°C.”
    - Does the spectrometer have an entrance aperture/slit? Or is the mentioned pinhole used as entrance aperture? What is its width?
    - Quite low temperature of -90°C, could you give a reason for this setting? Exposure times are stated with two seconds only.
- How did you validate during measurements, that you actually hit the azimuthal angles of 0° and 90°? Can you give a measurement uncertainty and is it considered during RCWA simulations or the influence on reconstruction accuracy?
- “By focusing on relative changes, the analysis becomes more straightforward and less prone to systematic errors.”
    - Systematic errors like the one on incidence angle, still should be non-negligible, so this statement at least is not correct in all accounts.
- “The experiments hereby presented were carried out with a 2s exposure time and by averaging over five spectra per dataset.”
    - Could you please give a statement on the statistics if only five spectra are used? Maybe sufficient if the source is running very stable.

Diffraction modelling

- Besides Diffraction of the different grating morphologies, was the reference reflectance of the unstructured sample also simulated to create the rel. reflectivity change and if so, also by RCWA?
- Figure 3
    - As the simulations were done in larger increments, isn’t the data shown here interpolated, and if so: What approach was used to interpolate between datasets? How is this considered during parameter reconstruction.
- Figure 4
    - Indicate linewidth and CD in the plots of Figure 4 for clarity.
- “Conversely, under conical diffraction conditions the diffraction efficiency profiles are largely independent of CD, as shown in the 3(d) […]”
    - Is that generally the case or only because you use radiation at a much smaller wavelength than the pitch/CD size? --> Less diffraction, less sensitivity. The sensitivity to height comes additionally from “thin film/Bragg interference” where your wavelength matches the probed height difference much better.
- “This is an intrinsic limit for 0th order scatterometry that affects the reconstruction precision of transverse features in general, that can only be overcome by collecting more

information from other diffraction patterns, such as the second orders for accurate CD reconstruction."
  - Can you really say that? See comment on independence between diffraction efficiency and CD.

Morphology reconstruction

- Least square regression:
  - Do I understand correctly, that you used the LSR only to compare experimental data with data from your prepared database. If so, how did you extract the morphology parameters, if they are set between two dataset points? (Related to the question on the interpolated (?) Plot in Figure 3.

Experimental results and discussion

- "Each peak was individually fitted to a Gaussian curve, and the area under the curve within the 1-sigma width was considered as the signal value for each datapoint."
  - Doesn't a peak in the HHG spectrum not have an intrinsic width, that is made of radiation with an according bandwidth? What is the bandwidth of your peaks? Is that then your wavelength accuracy? What would be the possible accuracy of your used spectrometer? Don't you lose important wavelength resolution by your method? What did you do, when using the HHG in the mode of quasi-continuous spectra?
- Did you only consider statistical uncertainties in your data? What about all possible systematic errors? (Angle accuracy, long-term source stability, straylight, etc. What about it's influence on reconstruction accuracy?
- Figure 5
  - In the figure caption the order between CD and groove height is switched for the plot in c), this could be easily adjusted.
- Figure 6
  - Could you comment on the visible multimodalities.
  - Why is AFM used here? I guess the cross-sectional SEM could give more realistic insight. Eitherway, how many lines have been considered to estimate the reference values?
- "This approach, however, cannot deliver accuracy in feature reconstruction better than the simulation mesh spacing, which was 1 nm for the groove height and 10 nm for the CD in the library used in this work."
  - 1.previously you wrote about spacings of 2nm / 25nm respectively. Which one is correct?
  - 2. If your accuracy cannot be better than this, why do you give smaller values in your plots in Fig. 6 & 7? Does the Std in the plots only come from taking the mean of all samples? I think that you have to expect variations between different samples (due to small fabrication variations) so taking this value as a measure for uncertainty is not reliable. Your first estimation on accuracy should be derived by the width of your euclidian distance distribution and only when that would be smaller than your data set steps, you can use these values as minimal accuracy.
- "The extra cleaning procedure proved effective in reducing the persistent resist layer, as characterized by means of XPS."
  - Was the contamination not detected already when you used XPS to identify the SiO-top layer of the samples? If not, why? Are all previous measurements used

for morphology reconstruction done with contaminated samples (in the supplements the cleaning step is named)?
If no, why bring this topic up so late and not beforehand?
If so, why couldn't the measurements und reconstruction be redone with cleaned samples (as it was done with the sample in fig.8 c)? (beamtime is an argument I guess)

References

- Lohr et al. is referenced twice (date is wrong one time).
- Number of references (52) does not match references in text (Number only goes up to 45)
- Reference 48 is just 'Optica Publishing group'

Supplementary

- "After the fabrication process, the samples were characterized via X-ray Photoelectron Spectroscopy which measured a 1.2±0.1nm thick native oxide layer on the surface."
    - The resist residues are not mentioned here, a bit confusing. How does this line-up in the storyline?
- Maybe add other/better SEM images.
- Maybe combine with some AFM scans.
